# Machine-driven parameter screen of biochemical reactions

**DOI:** 10.1093/nar/gkaa079

**Published:** 2020-02-06

**Authors:** Stéphane Poulain, Ophélie Arnaud, Sachi Kato, Iris Chen, Hiro Ishida, Piero Carninci, Charles Plessy

**Affiliations:** 1 RIKEN Center for Life Science Technologies, Division of Genomics Technologies, Yokohama, Japan; 2 RIKEN Center for Integrative Medical Sciences, Division of Genomic Medicine, Yokohama, Japan; 3 Biomedical Microsystems Lab., Institute of Industrial Science, The University of Tokyo, Tokyo, Japan; 4 Labcyte Inc., Tokyo, Japan; 5 Okinawa Institute of Science and Technology Graduate University, Genomics and Regulatory Systems Unit, Onna-son, Japan

## Abstract

The development of complex methods in molecular biology is a laborious, costly, iterative and often intuition-bound process where optima are sought in a multidimensional parameter space through step-by-step optimizations. The difficulty of miniaturizing reactions under the microliter volumes usually handled in multiwell plates by robots, plus the cost of the experiments, limit the number of parameters and the dynamic ranges that can be explored. Nevertheless, because of non-linearities of the response of biochemical systems to their reagent concentrations, broad dynamic ranges are necessary. Here we use a high-performance nanoliter handling platform and computer generation of liquid transfer programs to explore in quadruplicates 648 combinations of 4 parameters of a biochemical reaction, the reverse-transcription, which lead us to uncover non-linear responses, parameter interactions and novel mechanistic insights. With the increased availability of computer-driven laboratory platforms for biotechnology, our results demonstrate the feasibility and advantage of methods development based on reproducible, computer-aided exhaustive characterization of biochemical systems.

## INTRODUCTION

Systematic explorations of reaction parameters have been driven by automation and miniaturization of laboratory experiments, and sub-microliter liquid handling systems hold the biggest promises in terms of throughput together with reducing the cost of reagents. For instance, microfluidics technologies were used to generate 64 different combination of salt and DNA concentrations in a hybridization assay, for five different salts (1). More recently thousands of unique combinations of different concentrations of reagents and their reaction products were measured using microfluidic droplets (2). However, such approaches rely on merging physical streams of reagents, and thus are limited in the number of parameters that can be explored simultaneously and are also hard to apply to categorical parameters. On the other hand, several nanoliter-handling platforms have appeared on the market and empower researchers to design digitalized analysis with an arbitrary number of reagents. Among these platforms we chose acoustic droplet ejection technology because it combines several advantages. First, there is no contact between the machine and the liquid, which eliminates the cost of disposable plastic pipette tips. Second, source reagents can be provided in microplates with hundreds or even thousands of wells, allowing for the use of molecular barcodes. Third, each liquid transfer is fast.

**Figure 1.**
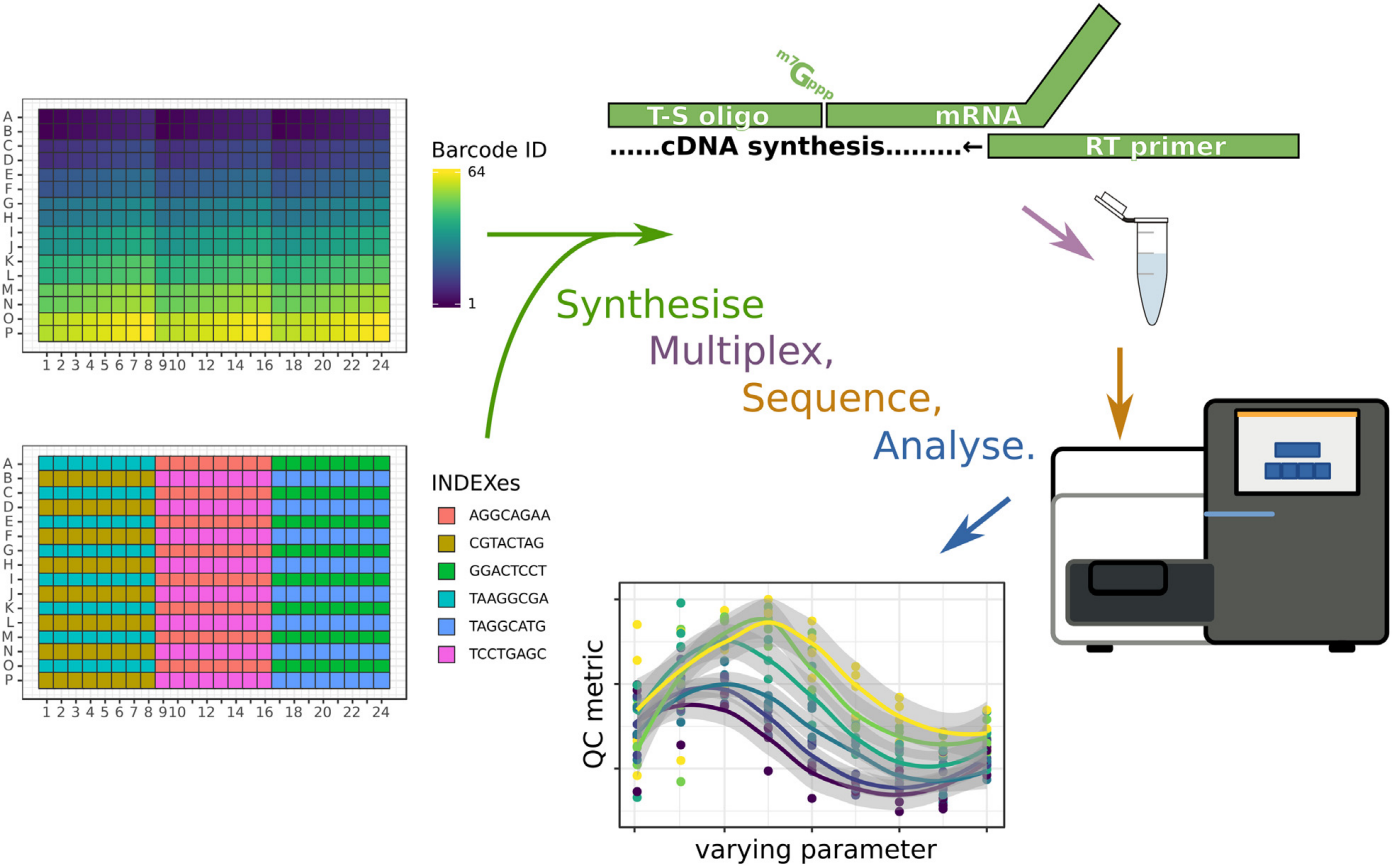
Method overview. This conceptual drawing shows a 384-well plate where each individual well is recognized by a combination of 64 different ‘barcodes’ and six different ‘indexes’. The studied reaction is a reverse-transcription where a mRNA is converted to cDNA by a reverse transcriptase (RT) using a primer. In addition, a template-switching (T-S) oligonucleotide is present in the reaction to extend the sequence of the mRNA. After incubation in the plates, reactions prepared with six different starting amounts of RNA are amplified together in six different polymerase chain reactions using indexed primers, pooled in a multiplexed sequencing reaction and then demultiplexed *in silico* using the unique combination of barcode and index sequences. Multi-factorial quantitative analysis of the sequencing reads then follows, according to the initial reaction parameters decided for each well in the experiment design.

As a pilot reaction for optimizing, we chose reverse-transcription, which converts messenger RNA (mRNA) molecules to complementary DNA (cDNA), a suitable substrate for quantitative DNA sequencing technologies (3). The reverse-transcription reaction is central to biological experiments that aim at quantifying the activity of genes. In experiments where the amount of biological substrate is limited in quantity, the performance of the reverse-transcription reaction becomes a limiting factor. In particular, analysis of single cells requires a highly efficient conversion from mRNA to cDNA (4). The reverse-transcriptase enzyme needs a short DNA oligonucleotide to prime the reaction. In addition, many methods for single-cell analysis use an additional ‘template-switching’ oligonucleotide in order to extend the cDNA sequence in preparation for sequencing (see Figure 1 and for review, Picelli, 2017 (5)). While increasing concentrations of the reverse-transcription primer and the template-switching oligonucleotide tend to increase the efficiency of the reaction, a limit is reached at high values (see for instance Zajac *et al.* (6)). The proliferation of protocols using clearly different reaction parameters (Table 1) suggests rather that oligonucleotide concentrations seen as optimal by protocol developers depend on other parameters such as enzyme type or substrate amounts.

**Table 1.** Comparison of reverse-transcription reagents concentrations used in different RNA sequencing protocols. TSO: template-switching oligonucleotide

TSO (nM)	RTP (nM)	dNTP (mM)	Mg (mM)	Mn (mM)	DTT (mM)	Enzyme (units)	Reference
10000	1000	2.5	3	0	10	SSIII (200)	Poulain 2017
1000	1000	2	7.2	0	4	SSII (50)	Lee 2017
4600	135	1.2	8.7	0	5.4	SSII (6.8)	Hochgerner 2017
1000	100 or 1	1	?	0	2.4	SmartScribe (100)	Turchinovich 2014
1000	1000	1	12	0	2.5	SSII (100)	Picelli 2013
200	200	1	3	3	1	SSII (5)	Islam 2011
0	750	4	3	2	5	SSII (200)	Schmidt 1999
0	65789	1.1	3	0	5	SSIII (760)	Murata 2014

RTP: reverse-transcription primer. The other reagents (desoxynucleotide triphoshates (dNTP), magnesium (Mg) and manganese (Mn) ions and dithiothreitol (DTT)) are listed for the sake of completeness but were not investigated in this manuscript.

In the past decade, we have developed a method for quantitative high-throughput gene expression analysis, nanoCAGE (7,15), in which template-switching oligonucleotides and random reverse-transcription primers are used to introduce adapters at both ends of the cDNAs (Figure 1) for amplification and sequencing. The key features of the nanoCAGE method are that its reverse-transcription primers have random ends to cover non-polyadenlyated RNAs such as histone mRNAs and non-coding RNAs, and that the riboguanosine tails of the template-switching oligonucleotides are used to increase the specificity for capped mRNA ends (15,16). While these two oligonucleotides are essential to the reaction, they are also a source of noise as for instance they can prime each other and create artificial templates for the PCR reaction that follows. Thus, the choice of their concentrations was a trade-off between reaction efficiency and reaction specificity.

We originally developed nanoCAGE for samples yielding nanogram amounts of total RNA input. In the latest version of the nanoCAGE protocol (7), the PCR-amplified cDNAs are fragmented and prepared for sequencing by tagmentation with the Tn5 transposase (17). As a barcode sequence is introduced at the 5′ end of cDNA molecules by the template-switching oligonucleotide during the reverse-transcription step and an index sequence is added at the 3′ end of sequencing products during the tagmentation step, several samples can be multiplexed and sequenced together. Additionally, Unique Molecular Identifiers (UMI) are also included in the sequence of template-switching oligonucleotides, which allows single molecule counting by removal of PCR duplicates in sequencing data. Since nanoCAGE is focused on 5′ ends and does not aim at full transcript coverage, libraries can be completely sequenced even on an Illumina MiSeq sequencer (see for instance Tauran *et al.* (18)).

In pilot experiments, we obtained evidence that the latest nanoCAGE protocol might be miniaturized for picogram amounts of total RNA, but it remained unclear whether the amount of the other reagents in the reverse-transcription would need to be scaled down. We routinely use six quality control metrics (Table 2) to assess the performance of nanoCAGE reactions. Importantly, these metrics require less than a thousand of reads per experiment to converge quickly to stable values, therefore allowing us to massively multiplex nanoCAGE reactions in small-scale, cost-efficient sequencing runs. Using nanoCAGE quality metrics as a proxy for the quality of reverse-transcription reactions, we developed the method presented here, to search for optima in the 4D parameter space representing enzyme type, RNA starting amount and molarity of the template-switching oligonucleotide and the reverse-transcription primer.

**Table 2.** Quality control metrics

QC metric	Explanation
Amount of oligonucleotide artefacts	Percentage of demultiplexed reads pairs that are discarded by the TagDust software with its low-complexity filter or by matching to a list of synthetic DNA adapter sequences. These removes artefacts such as PCR primer dimers and products of the reverse transcription of oligonucleotides by other oligonucleotides.
Amount of ribosomal RNA sequences	Percentage of demultiplexed read pairs that match reference ribosomal RNA sequences. Since ribosomal RNAs are very abundant, their sequencing is at the expense of the sequencing of other genes and therefore must be avoided to reduce the experimental cost.
Mapping rate	Percentage of demultiplexed read pairs that are ‘properly’ aligned to the genome (head to head on the same strand at a distance shorter than 2 Mb). This measures the amount of sequencing reads that is effectively spent measuring gene expression levels.
Promoter rate	Percentage of properly mapped reads that are aligned in promoter regions, arbitrarily defined as windows of 1000 nucleotides centered on start positions of GENCODE M1 transcripts.
Strand invasion rate	Percentage of molecule counts that aligned directly downstream genomic regions matching oligonucleotide sequences, as described in Tang *et al.*, 2013. These artefacts, detected after mapping and molecule counting (PCR deduplication), cause mischaracterization of promoters and bias the measurement of their activity
Richness	Richness score (Hurlbert 1971), representing the expected number of molecules that would align in known genes if the library were downsampled to 10 reads.

## METHODS

**Figure 2.**
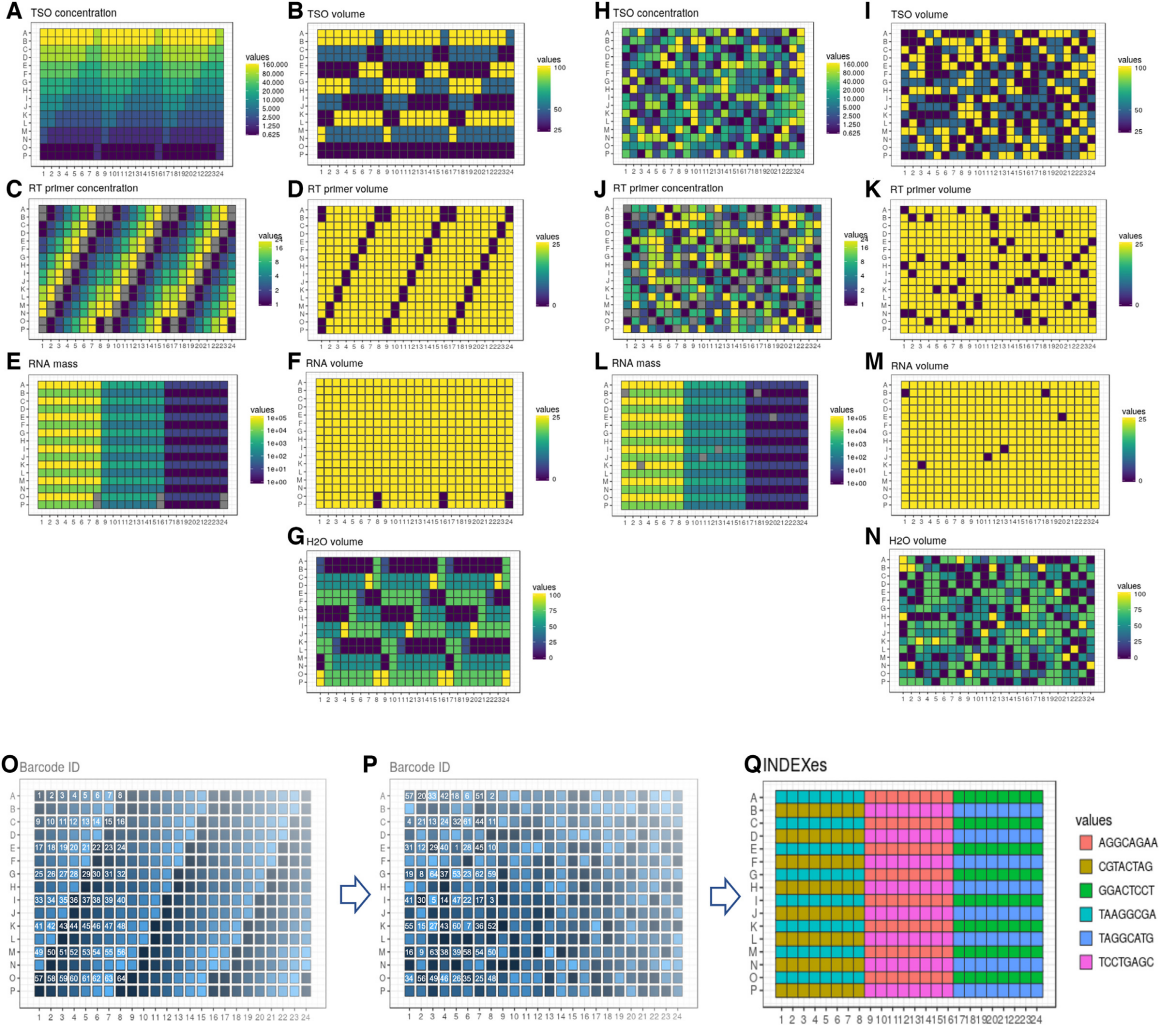
Randomization of nanoCAGE reverse transcription reactions. (A–N) Distribution of reagent concentrations (**A, C, E, H, J, L**) and related liquid transfer volumes (**B, D, F, G, L, K, M, N**) in a 384-well plate before (A–G) and after randomization (H–N). Primer concentrations are displayed on a mM scale (9 different TSO concentrations ranging between 0.625 and 160 mM, and six different RT primer concentrations ranging between 1 mM and 24 mM), RNA masses are shown on a picogram scale (6 different RNA quantities ranging from 1 pg to 100 ng) and reagent transfer volumes are indicated on a nanoliter scale. Wells displayed in gray correspond to 60 negative control reactions performed without RT primers (see C and J) or without RNA (see E and L). There were thus 324 reagent combinations studied on each plate using two different enzymes (SSIII and SSIV), which represents a total of 648 data points used to explore the parameter space of the reaction. (O–P) Example of reverse transcription barcode ID distribution before (**O**) and after randomization (**P**) for a set of 64 reactions corresponding to one RNA mass (wells indicated in yellow on E and L). The 64 barcoded cDNA samples obtained after the reverse transcription were pooled, amplified and tagged by a specific index sequence (**Q**) representative of the corresponding group of samples in the sequencing library. Six different indexes characteristic of the six RNA masses were used for each plate. Randomized plates were prepared in quadruplicate for each reverse transcriptase using 24 different indexes, making a total of 1536 samples (64 barcoded samples x 24 indexes) that were pooled and sequenced together for each enzyme.

Nanoliter-scale liquid transfers were performed on an Echo 525 instrument (Labcyte) using transfer sheets generated from event logs produced by simulations of instrument runs in the R programming language. All the scripts are archived as supplemental material on Zenodo and available for display at https://github.com/oist/labcyte-rt-optimisation/tree/1.0.0. Each 384-well plate was organized in six zones interleaved to match the geometry of standard multichannel pipettes in order to collect the contents of their wells and pool them in microtubes (Figures 1 and 2). Within a zone, each of the 64 wells was recognized with a unique barcode sequence inserted in the template-switching oligonucleotide. The barcoded oligonucleotides were chosen randomly from a list of 70 (out of 96) that passed quality controls (see below). Each zone received 9 × 6 combinations of molarities of template-switching oligonucleotides and reverse-transcription primers, plus nine controls with no reverse-transcription primers, plus 1 control with no RNA (Figure 2). Wells from each zone received a different amount of total RNA. Four plates were prepared with the SuperScript III enzyme (Invitrogen) and four with SuperScript IV (Invitrogen).

We used a total RNA prepared from mouse liver. An RNA Integrity Number (RIN (19)) of 9 was measured on an Agilent Bioanalyzer with an RNA 6000 pico kit. As molarity measurement were not possible with this kit, RNA amounts are measured as a mass in this work.

**Figure 3.**
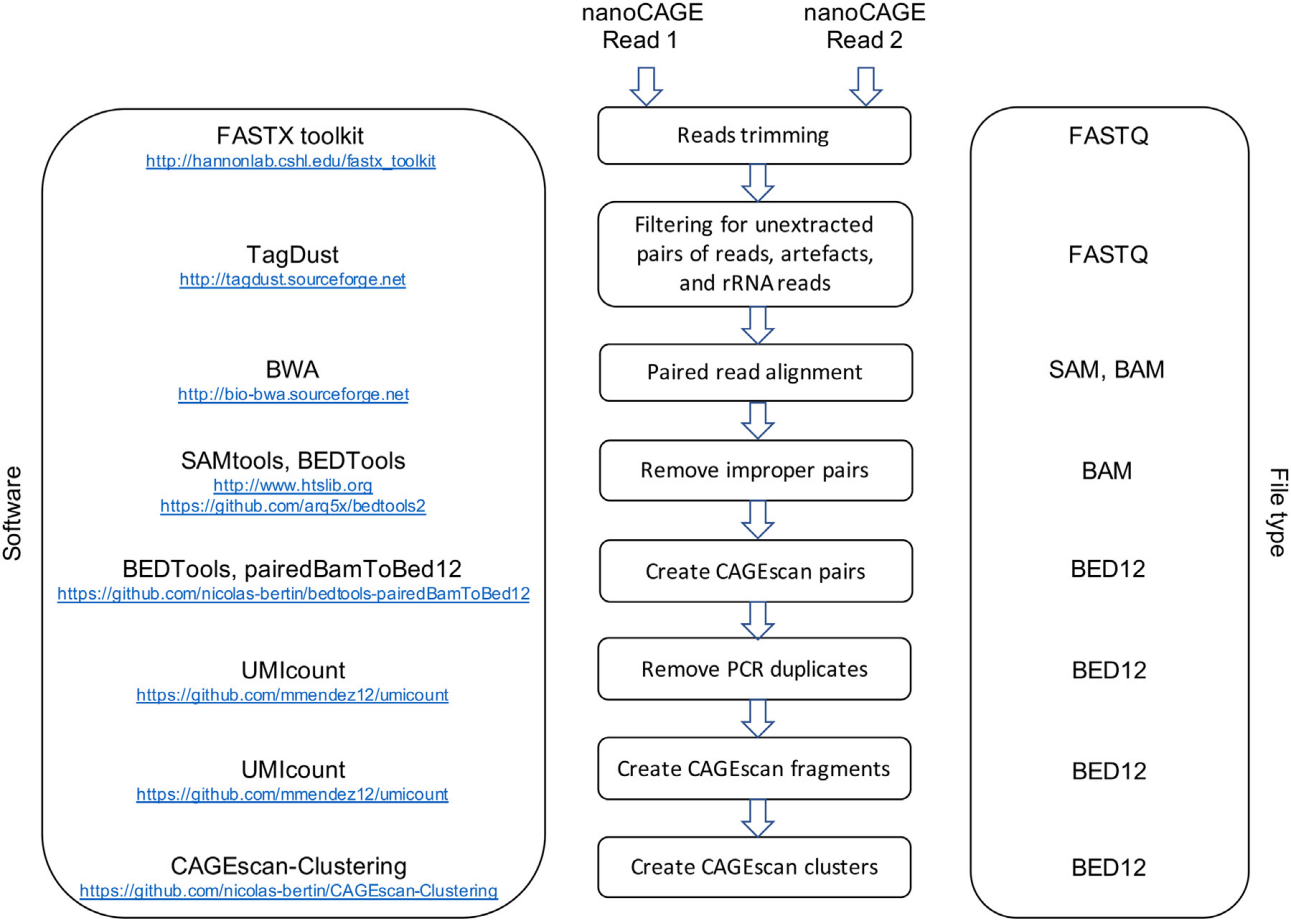
Block diagram summarizing the MOIRAI workflow used to process the sequencing data. The full data for each sequencing run has been deposited to ZENODO (https://doi.org/10.5281/zenodo.1683162).

The template-switching oligonucleotides were synthesized by Integrated DNA Technologies, Inc. (IDT), at a scale of 10 nmol each in a 96-well plate format and resuspended at a stock concentration of 1 mM upon reception. To assess the quality of oligonucleotide synthesis, we ran a control experiment in which the parameters of the reverse-transcription reactions were kept constant (see supplemental experiment six on https://github.com/oist/labcyte-rt-optimisation/blob/1.0.0/Labcyte-RT_Data_Analysis_6.md for details). For the main experiment, we selected 70 oligonucleotides among those who gave sequence yields closest to the mean. RT reaction master mixes (containing 0.0528 M sorbitol, 0.264 M trehalose, 0.75 M betain, 0.01 M DTT, 0.625 mM dNTPs, 20 U/μl SuperScript enzyme III or IV and 1× SuperScript buffer III or IV), template-switching oligonucleotides, reverse-transcription primers, RNA and ultrapure water were transferred from independent wells of a source plate (Greiner Bio-One) to the different wells of the destination plates (Applied Biosystems) using specific liquid transfer patterns for each plate. After the acoustic transfer of RT reagents (total RT reaction volume equal to 500 nl), the 384-well destination plates were directly sealed, centrifuged at 4°C, and deposited in a 7900HT Fast Real-Time PCR system (Applied Biosystems) to perform first strand cDNA synthesis (temperature program: 22°C for 10 min; 50°C for 30 min; and 70°C for 15 min). RT products from each zone (see above and Figure 2) were further collected with a multichannel pipette, pooled and purified using AMPure XP beads (Beckman Coulter). Purified pools were then processed as in the nanoCAGE protocol (7) applying 18, 21, 24, 27, 30 and 33 PCR cycles during the first amplification reaction, respectively for pools of RT reactions corresponding to template RNA amounts ranging from 100 ng to 1 pg. Libraries prepared for each starting RNA amount were tagged by specific index sequences (Nextera XT DNA Library Preparation Kit, Illumina), mixed equimolarly and sequenced on a MiSeq system using a reagent kit v2 (Illumina) with 46 cycles for the first read, 8 cycles for the index and 21 cycles for the second read. One run was performed for SuperScript III and one for SuperScript IV, reusing the same indexes. Sequencing data were processed with the MOIRAI workflow system (20) (Figure 3) to produce alignment files (BED), which were loaded into R using the CAGEr Bioconductor package ((21), https://bioconductor.org/packages/CAGEr). Sequencing quality metrics (Table 2) were calculated using custom R scripts (see https://github.com/oist/labcyte-rt-optimisation/blob/1.0.0/Labcyte-RT_Data_Analysis_8.md for details). Richness indexes were calculated on gene expression levels (mapped counts per gene) using the ‘rarefy’ function of the ‘vegan’ R package (22). Variability of the replicates was assessed by calculating the mean average difference (MAD) and normalizing it by dividing it by the mean. Its value was lower than 0.2 in more than half of the replicates. Contour plots of the normalized MAD, as well as standard plots displaying all replicates of each parameter combination are available as supplemental figures.

For single cell experiments, a suspension of HeLa cells (ATCC ref. CCL-2) was prepared, filtered through a 40 μm cell strainer (Falcon), and sorted by flow cytometry (FACSAria II, BD Biosciences) in order to transfer individual cells into 96-well plates (Applied Biosystems). After single cell isolation, the plates were directly sealed, centrifuged at 4°C, and stored at −80°C until further processing. Cell lysis was initiated upon thawing frozen plates on ice for 15 min. Next, RT master mixes (containing 0.0528 M sorbitol, 0.264 M trehalose, 0.75 M betain, 0.01 M DTT, 0.625 mM dNTPs, 1 μM or 0.4 μM RT primers, 20 U/μl SuperScript enzyme III or IV and 1× SuperScript buffer III or IV) were transferred with a multichannel pipette. Specific barcoded template-switching oligos (10 or 45 μM) were then added in each well according to the multiplexing plan. After the addition of RT reagents (total RT reaction volume equal to 4 μl), the plates were directly sealed, centrifuged at 4°C and placed in a 7900HT qPCR system to carry out first strand cDNA synthesis (temperature program for SuperScript IV: 22°C for 10 min; 50°C for 15 min; and 80°C for 10 min; temperature program for SuperScript III: 22°C for 10 min; 50°C for 30 min; and 70°C for 15 min). RT products were harvested with a multichannel pipette, pooled and purified using Ampure XP beads (Beckman Coulter). Purified pools of single cell RT products were processed for nanoCAGE library preparation (7). Pools of reactions performed using standard primer concentrations with SuperScript III or SuperScript IV; and using alternate primer concentrations with SuperScript IV were subsequently tagged by specific index sequences (Nextera XT DNA Library Preparation Kit, Illumina), pooled and sequenced on a MiSeq instrument. Sequence data were subsequently processed with MOIRAI and custom R scripts as described above.

## RESULTS

Using a Labcyte Echo 525 instrument, we assembled 500 nl reverse-transcription reactions in 384-well plates, by dispensing droplets of 25 nl from a source plate containing the reagent stocks to the destination plates. After incubating the reactions, we assessed their yield and quality by quantitative DNA sequencing using the nanoCAGE method. We sequenced two multiplexed pools of reactions using a selected set of 64 different template-switching oligonucleotides carrying sample identifiers (‘barcodes’), combined with a second set of 24 sample identifiers (‘indexes’) (Figure 2). Each pool contained 1536 reactions, prepared in four plates. As we focused on quality controls, the sequencing was kept at a small scale: ∼5000 sequence read pairs per reaction.

We explored one categorical and three continuous parameters spanning multiple orders of magnitude with data points regularly spaced on a logarithmic scale. The broadest range was for RNA amounts, with 6 points ranging between single-cell amounts (1–10 pg) and starting amounts typical for bulk RNA libraries (10–100 ng, or a few μg in larger reaction volumes). The second broadest range was the molarity of the template-switching oligonucleotide (nine points between 0.6 and 160 μM), followed by the reverse-transcription primer's molarity (six points between 1 and 24 μM). Combined with a categorical dimension encoding two different reverse-transcription enzymes: SuperScript III (SSIII) and SuperScript IV (SSIV), we thus generated 648 different combinations, to which were added 120 negative controls. We prepared four replicates, each of which using two plates (one per enzyme).

To prevent position biases in the reaction microwell plates, such as drying on the edges or inhomogeneous heating during reaction, we randomized the coordinates of the reactions in each replicate (Figure 2). This randomization also has the effect of making each replicate plate unique, which prevents from accidental swapping. In particular, the different positions of the negative controls in each plate act as an identifying fingerprint. Lastly, since we used barcoded oligonucleotides, the randomization prevented that barcode sequences, individual reagent quality, or position-specific variability in the reagent transfer would get confounded with any reaction parameter. Randomizations were made entirely reproducible by the computer-aided generation of machine-readable volume transfer instructions and were reproducibly repeated for each replicate with a different random seed.

**Figure 4.**
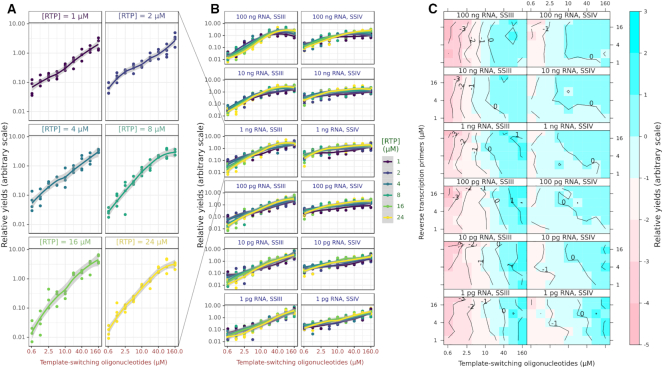
Parameter screen at high dynamic range. (**A**) Log-scaled relative yields of demultiplexed read counts (in arbitrary units) as a function of the molarity of the template-switching oligonucleotide, for different molarities of the reverse-transcription primers (RTP), using the SSIII enzyme and 100 pg RNA. (**B**) Same data displayed for all RTP molarities, colour-encoded as a categorical variable, and the SSIII and SSIV enzymes. In this panel, each replicate is displayed as a dot. (**C**) Median relative yields of demultiplexed read counts (in arbitrary units) over each set of four replicates, displayed as a contour plot on a surface where each axis represents the molarity of one oligonucleotide. The colour scale indicates better (blue) or worse (red) performance.

To ensure equal sequence coverage, the PCR amplifications that followed the reverse-transcriptions were scaled according to the starting quantities of RNA. Each PCR amplification was multiplexed with a different index (Figure 1). For each sample amplified in the same PCR reaction, we then calculated relative sequence yields, and observed a positive correlation with the molarity of the template-switching oligonucleotide (Figure 4A), and little effect of the reverse-transcription primer, except at the highest concentrations of both oligonucleotides and the RNA (Figure 4B). To better represent the effect of the interaction between the two oligonucleotides, we calculated the medians of each quadruplicate, and represented them as a contour plot (Figure 4C) on surfaces where the molarity of each oligonucleotide is one dimension. We use the same graphical representation for other quality control statistics in the remaining figures of the manuscript. As these plots do not show directly the variability between replicates, categorical plots versions in the same style of Figure 4B are available in the supplementary material.

**Figure 5.**
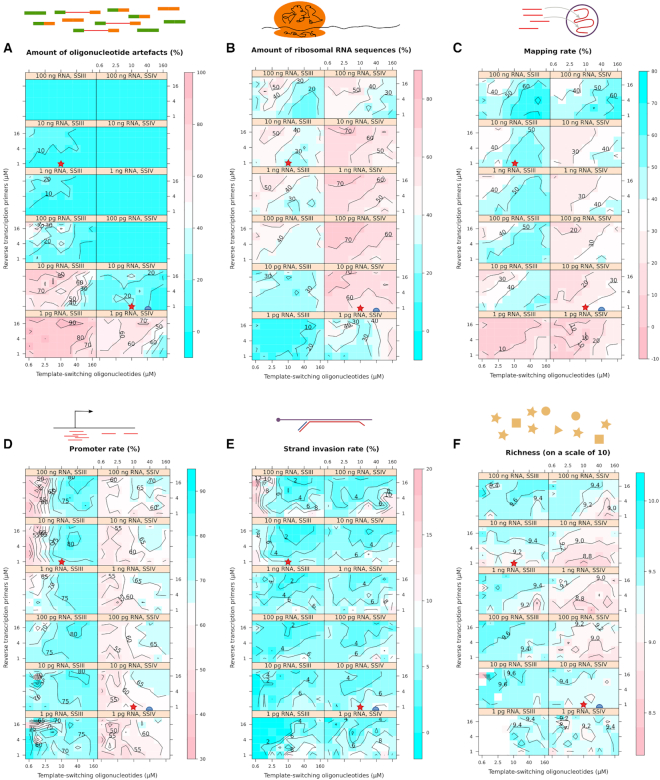
Quality and specificity across the parameter space. (A–E) percentage of (**A**) oligonucleotide artefacts, (**B**) reads mapping to reference ribosomal RNA sequences, (**C**) reads mapping to the genome, (**D**) reads aligning to promoter regions, (**E**) premature ‘strand invasion’ artefacts of the template switching reaction. (**F**) Richness index. The colour scale indicates better (blue) or worse (red) performance. Stars indicate oligonucleotide molarities of the standard nanoCAGE protocol. Half-circles indicate molarities of the modified protocol tested on single cells.

To assess the quality and specificity of the reactions, we then calculated metrics reflecting the suitability of the reactions for transcriptome analysis (Table 2, Figure 5). With RNA inputs lower than 100 pg, the SSIII enzyme started to produce large amounts of oligonucleotide artefacts, at a scale that would compromise its use in transcriptome analyses. The SSIV enzyme performed comparatively well on one order of magnitude lower RNA input (10 pg). For both enzymes, increasing the molarity of the reverse-transcription primer lead to an increase of the oligonucleotide artefacts, but this could be compensated by a proportional increase of the molarity of the template-switching oligonucleotide, as shown by the diagonal patterns on the contour plots (Figure 5A). A similar diagonal pattern can be observed in the contour plots for the ribosomal RNA rate (Figure 5B), showing again a positive effect in terms of quality for the reduction of the reverse-transcription primer's molarity and the increase of the template-switching oligonucleotide, especially for the SSIV enzyme. Another interesting trend was that overall, ribosomal RNA rates were lower for two extreme amounts of starting RNA material (100 ng and 1 pg). For SSIII at all RNA amounts and for SSIV at low RNA amounts, the highest molarities of the template-switching oligonucleotide were also increasing the ribosomal RNA rate. We then calculated the mapping rate, in which the ribosomal RNA sequences are considered unmapped. The contour plots (Figure 5C) were essentially the reverse of Figure 5B, showing that most non-ribosomal reads mapped correctly.

Within the set of all mapped reads, we calculated the promoter rate (Figure 5D). Both enzymes showed similar trends except that SSIV had a lower baseline. Increases of the molarity of the template-switching oligonucleotide again increased reaction quality except at the highest molarities. Strikingly, the best promoter rates were obtained at high molarity of the reverse-transcription primer, which is the opposite trend in comparison with the other quality metrics calculated above. All RNA amounts benefited from high oligonucleotide molarities.

We then calculated the proportion of mapped reads that could be strand-invasion artefacts (Figure 5E), which form when the template-switching oligonucleotide prematurely hybridizes with the nascent cDNA (23). Contrarily to the intuition that higher concentrations of this oligonucleotide would lead to higher frequencies of artefacts at higher molarity of the template-switching oligonucleotide, our results show a more complex relationship. For instance, the most extreme strand-invasion rates were reached with SSIII at high RNA amounts and low template-switching oligonucleotide molarity. This suggests that under these conditions, the artefacts may be more easily created than the desired products, and that higher concentrations of template-switching oligonucleotide and reverse-transcription primer are necessary to repress artefact formation. Thus, our approach gave us not only a fine-grained mapping of the optimal reaction conditions in the parameter space, but also mechanistic insights.While our strategy of shallow sequencing allows us to calculate accurate quality metrics despite a very low coverage of each reaction, it is impossible to compare the expression profiles with each other, or to know the total number of genes that would be detected if we had orders of magnitude deeper coverage. To estimate the potential for gene detection, we calculated richness indexes for each library following the method of Hurlbert (24), on a scale of 10 (Figure 5F). These indexes are the expected value (in the statistical sense) of the number of detected genes if the libraries were down-sampled to 10 mapped reads. In our libraries, surprisingly, richness indexes were higher at lower template-switching oligonucleotide molarities and higher reverse-transcription primer molarities. While intuitively it seems desirable to have higher richness indexes, it remains to be determined if they might be the reflection of a compression of the dynamic range.

**Figure 6.**
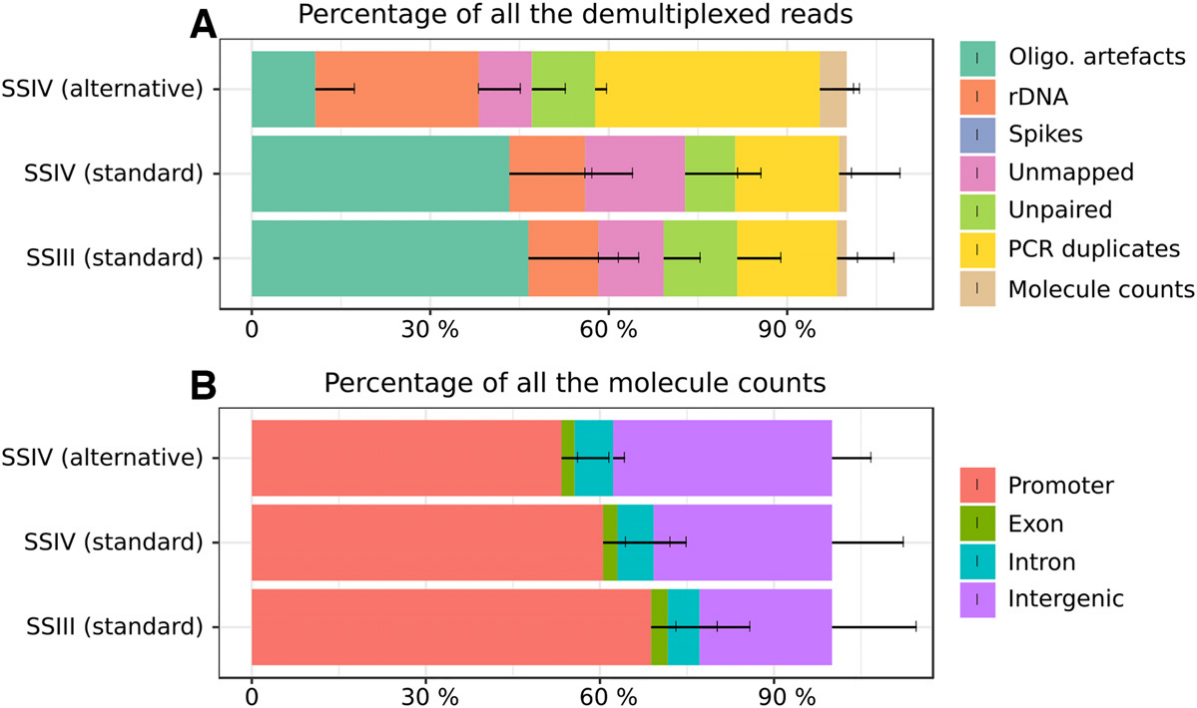
Test of alternative protocol on low RNA inputs (single cells). Stacked barplots summarizing the quality control statistics for single cell libraries made with the standard nanoCAGE protocol (SSIII standard, *n* = 136), or with the SSIV enzyme and the standard oligonucleotide concentrations (SSIV standard, *n* = 184), or with the SSIV enzyme with 45 μM template-switching oligonucleotide and 0.4 μM reverse-transcription primer (SSIV alternative, *n* = 83). (**A**) proportions of the reads discarded during data processing until obtaining unique molecule tag counts. (**B**) annotation statistics of the aligned molecule tags.

Our results confirm that bulk and single-cell reactions have different optima. At high amounts of starting RNA (100 ng), the standard nanoCAGE protocol (SSIII enzyme, 10 μM template-switching oligonucleotide, 1 μM reverse-transcription primer) is close to the optimal results, minimizing the amount of oligonucleotide and ribosomal RNA artefacts and maximizing the amount of data that correctly aligned to promoter regions. On the other hand, our results suggest that a protocol can be designed for single cells by using the SSIV enzyme, increasing the molarity of template-switching oligonucleotides and reducing the molarity of the reverse-transcription primers. Indeed, we validated this observation during the development of a single-cell version of the nanoCAGE protocol, where we tested the use of SSIV with the template-switching oligonucleotide molarity increased to 45 μM and the reverse-transcription primer molarity decreased to 0.4 μM, on single cells isolated by flow cytometry in 4 μL reverse-transcriptions reactions. As the amount of RNA per cell is usually estimated to be in the order of magnitude of 10 pg, the reaction concentration is in the order of the 1 pg / 500 nL condition in our parameter screen. In line with the screen's results, the change of enzyme and oligonucleotide concentrations dramatically reduced the amount of oligonucleotide artefacts, although at the expense of a reduced promoter rate and an increased rRNA rate (Figure 6).

## DISCUSSION

To our knowledge, we are the first to report the exploration of a four-dimension parameter space for a biochemical reaction using 648 data points. Our experimental design described here follows a systematic and exhaustive approach testing all combinations (‘grid search’) between a few parameters. Nevertheless, such a design cannot be extended to a larger number of parameters, because their total number of combinations would not fit into 384-well plates (although it would take advantage of smaller formats such as 1536-well plates or miniaturized array), and because high-dimension spaces are typically sparse. More efficient search strategies, such as random search (25), may enhance the power of the method. On the side of evaluation metrics, high dimensionality is also a problem. One possible workaround would be to reduce to two dimensions, for instance by calculating one arbitrary score representing undesirable products (oligonucleotides artifacts, ribosomal RNA sequences and strand invasion artifacts) and one arbitrary score representing efficient discovery of transcription start sites (mapping rate, promoter rate and richness), and then search for ‘Pareto efficient’ pairs representing states where the score on one dimension can not be ameliorated without deteriorating the score on the other dimension.

We observed a dramatic difference between the two reverse-transcriptases. According to the maker, SSIV has better thermostability, processivity and yield than SSIII. However, it is important to note that their reaction buffers also differ: SSIV’s buffer is proprietary and is advertised to perform better in the presence of inhibitors. Swap experiments would be needed for determining whether the enzyme or the buffer are the key factor driving our observations. Another simple explanation could be that the unit definition, which is ‘the amount of enzyme required to incorporate 1 nmole of deoxyribonucleotide into acid-precipitable material in 10 min at 37°C using poly(A) oligo(dT)_12–18_ as a template/primer’ may correspond to different actual quantities of enzyme, given their different thermal optima. Here, we compared two enzymes, but the number of reverse-transcriptases on the market is much larger (4). We expect differences at least as large as the ones observed between SSIII and SSIV. Our approach for a quantitative exploration of a reaction's parameter space may provide a method to better understand the impact of protein sequence differences between these enzymes, in particular between the engineered derivatives of the Moloney murine leukaemia virus reverse-transcriptase, provided that this information is disclosed by the vendors. Beyond addressing such questions, the approach that we have developed here will also provide the opportunity to search for ad-hoc mixtures of enzymes, and possibly buffers, that may maximize the output of the reaction (conversion to cDNA) and its quality (efficiency and specificity of template switching).

The reverse-transcription reaction that we studied here uses so-called ‘random’ primers that end with six random bases, for an even coverage of the transcriptome. Their drawback is the creation of artefacts when they hybridize to other oligonucleotides. In line with this, we observed that increasing the molarity of these reverse-transcription primers was increasing the amount of artefacts in the sequencing libraries (Figure 5A). Unfortunately, an opposite trend was seen on other quality statistics, such as the promoter rate (Figure 5D). This conflict may be hard to resolve. There are alternatives to random primers, such as oligo-dT primers that target the poly-A tail of the mRNAs, not-so-random primers (26) or pseudo-random primers (27). While the discussion of their pros and cons is beyond the scope of this manuscript, it is important to note that their optimal molarities are likely to be different.

Methods in molecular biology are made of a large number of serial steps, and the approach that we followed here is not limited to the reverse-transcription reaction. Here, we used quantitative sequencing for the readout, and this strategy can also apply to other reactions. For instance, the activity of a DNA-cutting enzyme (for instance Cas9) can be assayed by the degradation of a sequencing template. Conversely, the activity of a DNA-joining enzyme (for instance T4 DNA ligase) can be assayed by the assemblage of a template. Other platforms for the readout can be used, such as fluorescence detection, obviously for quantitative polymerase chain reaction, but also for any other reaction that can be designed to produce or degrade a fluorescent reporter.

Beyond the example presented here as a proof of principle, we believe that parameter space screening may become a routine experiment in the future. In particular, it will greatly benefit from computer-aided experiment design and robotic automation of experiment execution (28,29). The combination of laboratory automation and systematic parameter screening will ease the way to cross-replication studies in independent laboratories, as a strategy for cost-sharing, removal of implementation bias, and detection of human errors or data tampering.

## DATA AVAILABILITY

nanoCAGE sequence data: Zenodo 1680999 (https://doi.org/10.5281/zenodo.1680999). nanoCAGE sequence alignments: Zenodo 1683162 (https://doi.org/10.5281/zenodo.1683162). Single-cell sequence data: Zenodo 250156 (https://doi.org/10.5281/zenodo.250156). Single-cell sequence alignments: Zenodo 3340196 (https://doi.org/10.5281/zenodo.3340196). Source code for data analysis: Zenodo 3540405 (https://doi.org/10.5281/zenodo.3607187).

## Supplementary Material

gkaa079_Supplemental_File
