# Supplementary material for: Machine-driven parameter screen of biochemical reactions
Source: Nucleic Acids Res. 2020 Feb 6;48(7):e37. doi: 10.1093/nar/gkaa079 (PMC7144897; doi:10.1093/nar/gkaa079)
Supplement: gkaa079_Supplemental_File [file gkaa079_supplemental_file.pdf]

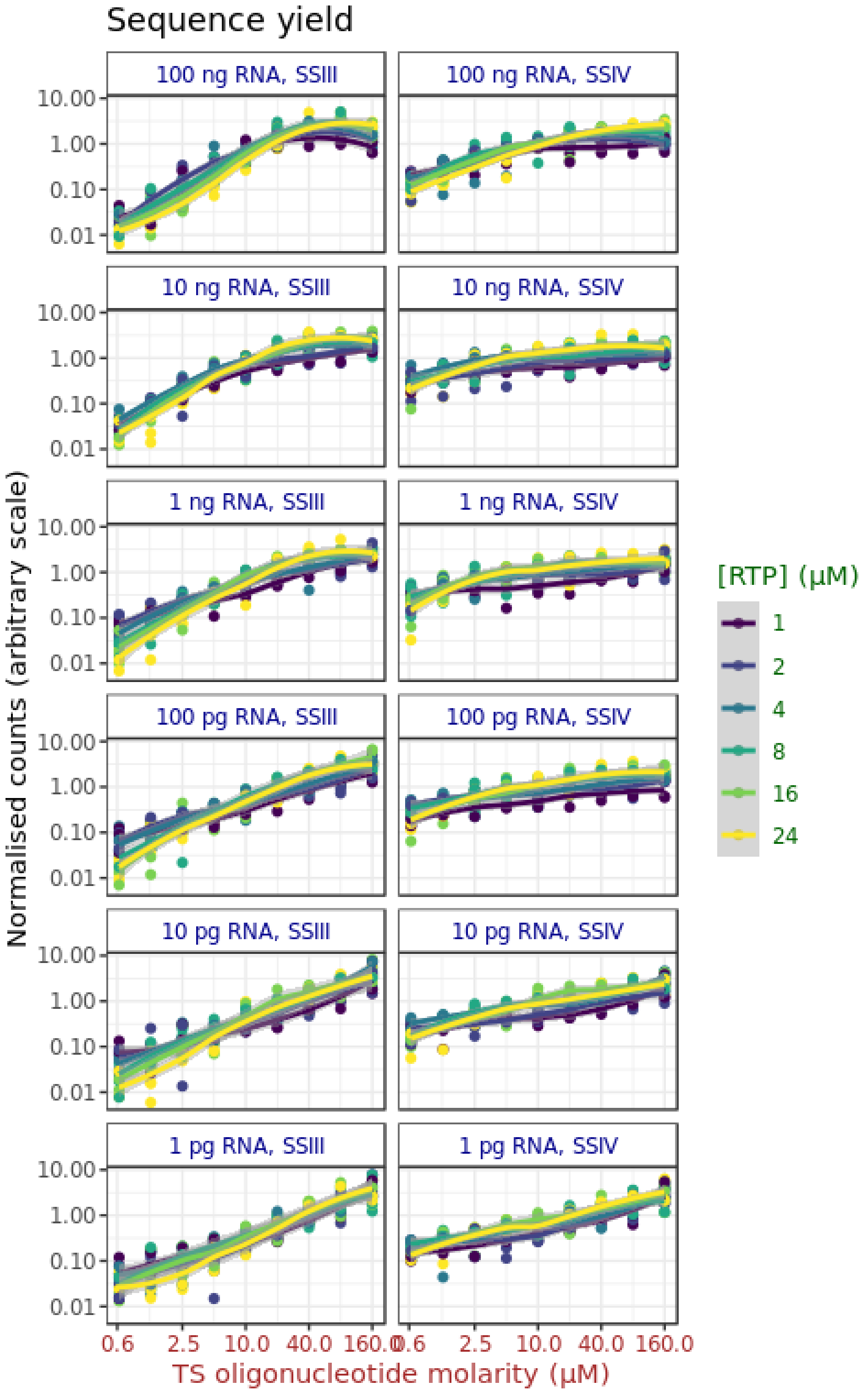

Sequence yield
100 ng RNA, SSIII
100 ng RNA, SSIV
10 ng RNA, SSIII
10 ng RNA, SSIV
1 ng RNA, SSIII
1 ng RNA, SSIV
100 pg RNA, SSIII
100 pg RNA, SSIV
10 pg RNA, SSIII
10 pg RNA, SSIV
1 pg RNA, SSIII
1 pg RNA, SSIV
10.00
1.00
0.10
0.01
Normalised counts (arbitrary scale)
0.6
2.5
10.0
40.0
160.0
TS oligonucleotide molarity (μM)
[RTP] (μM)
1
2
4
8
16
24

Amount of oligonucleotide artefacts

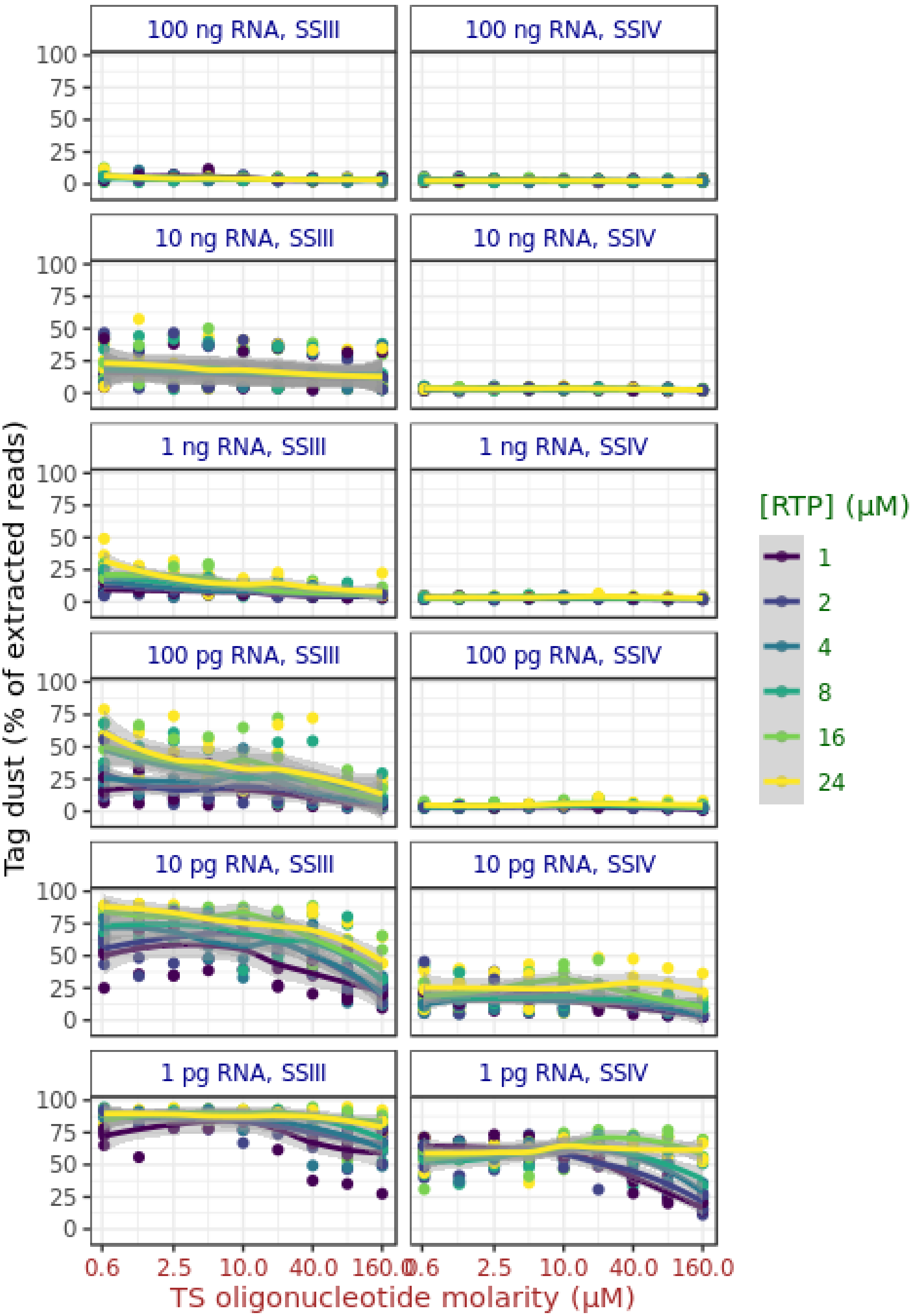

The amount of artefacts detected by TagDust is increased by RT primers and decreased by RNA and TSOs. SuperScript IV generated less artefacts than SSIII.

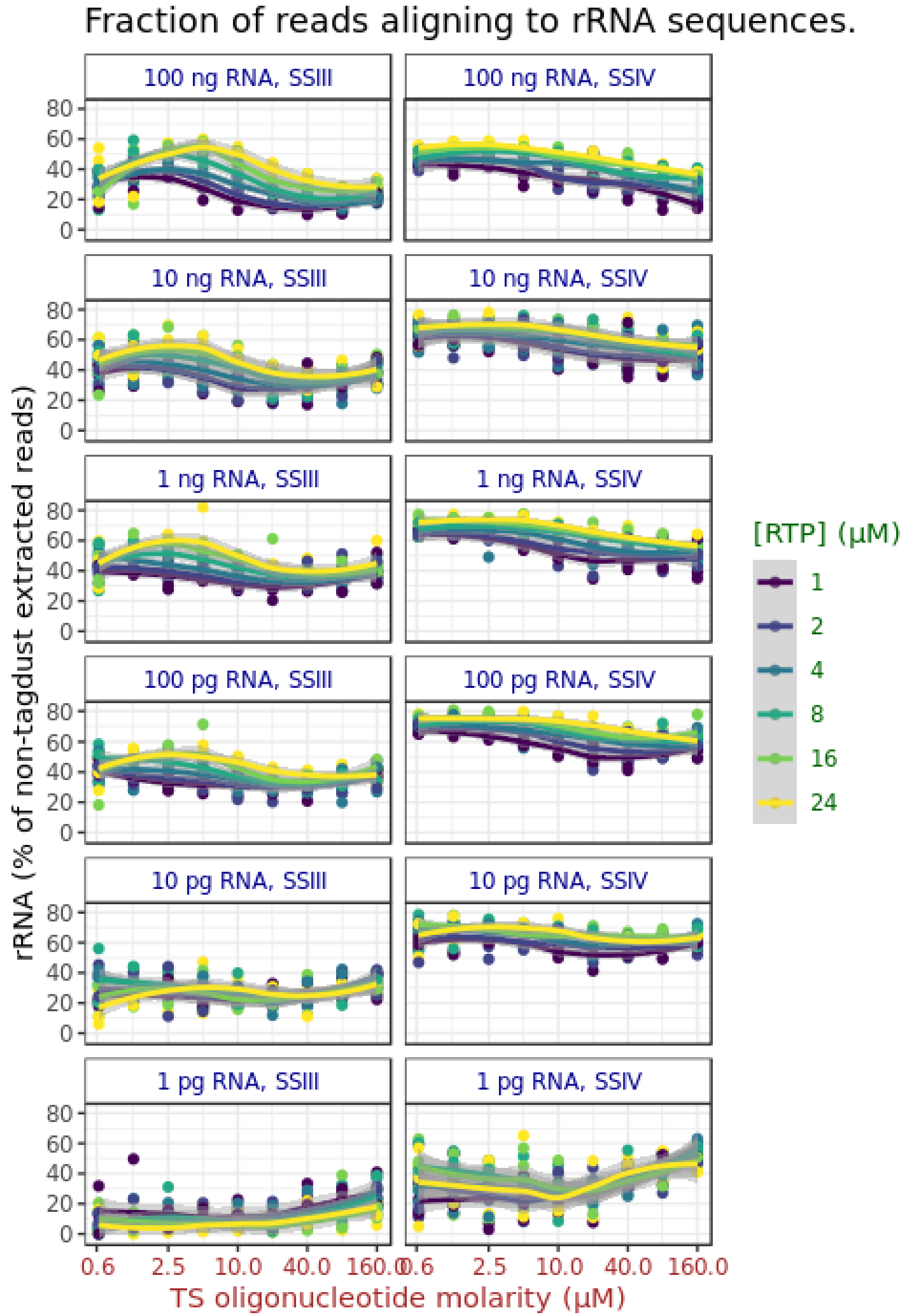

The fraction of reads aligning to ribosomal RNA (rRNA) sequences (after removing oligonucleotide artefacts) is increased by RT primers. It varies with TSO and RNA amounts. Overall, SuperScript IV gives more rRNA reads than SSIII.

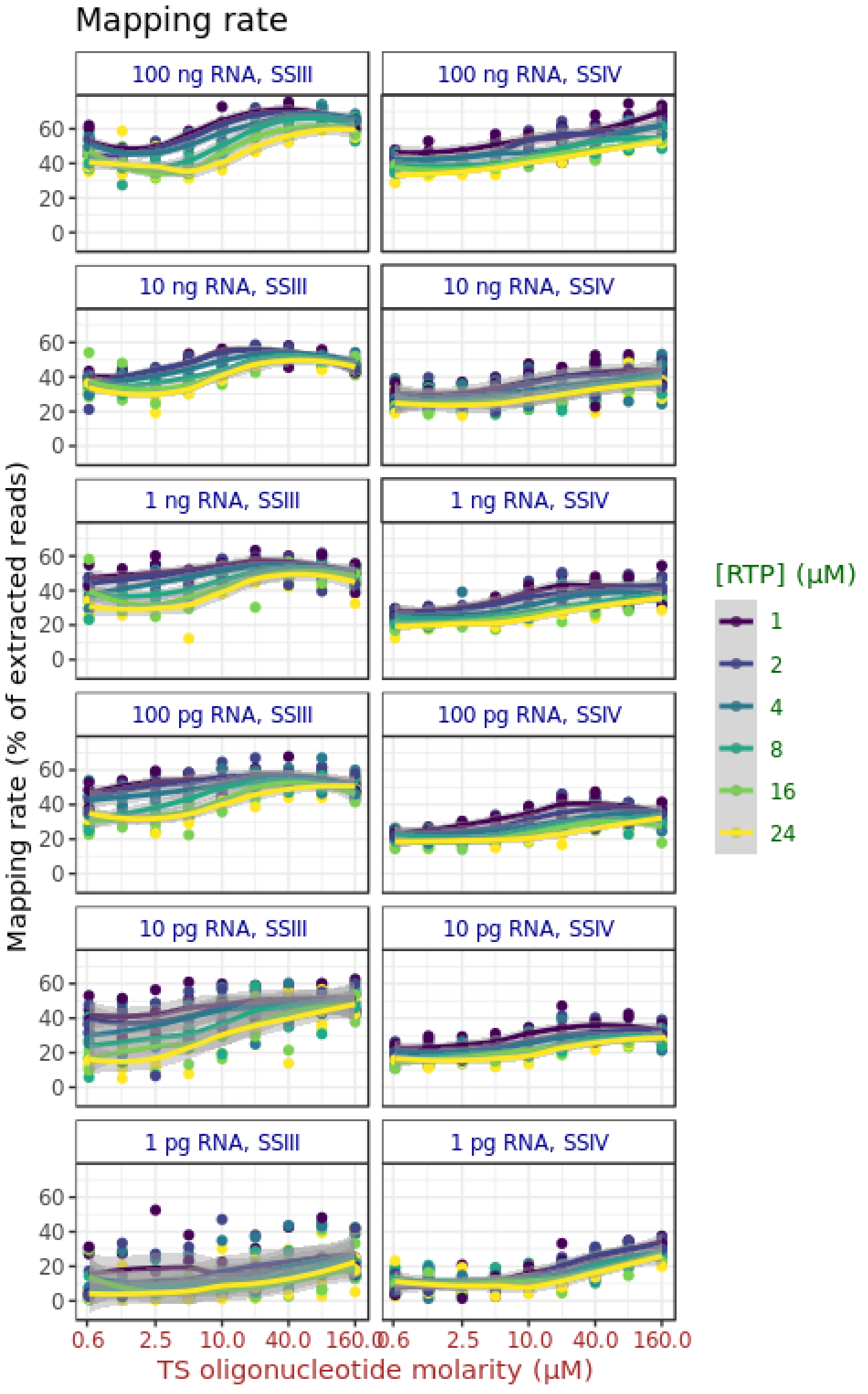

Mapping rate
100 ng RNA, SSIII
100 ng RNA, SSIV
10 ng RNA, SSIII
10 ng RNA, SSIV
1 ng RNA, SSIII
1 ng RNA, SSIV
100 pg RNA, SSIII
100 pg RNA, SSIV
10 pg RNA, SSIII
10 pg RNA, SSIV
1 pg RNA, SSIII
1 pg RNA, SSIV
0
20
40
60
Mapping rate (% of extracted reads)
0.6
2.5
10.0
40.0
160.0
TS oligonucleotide molarity (µM)
[RTP] (µM)
1
2
4
8
16
24

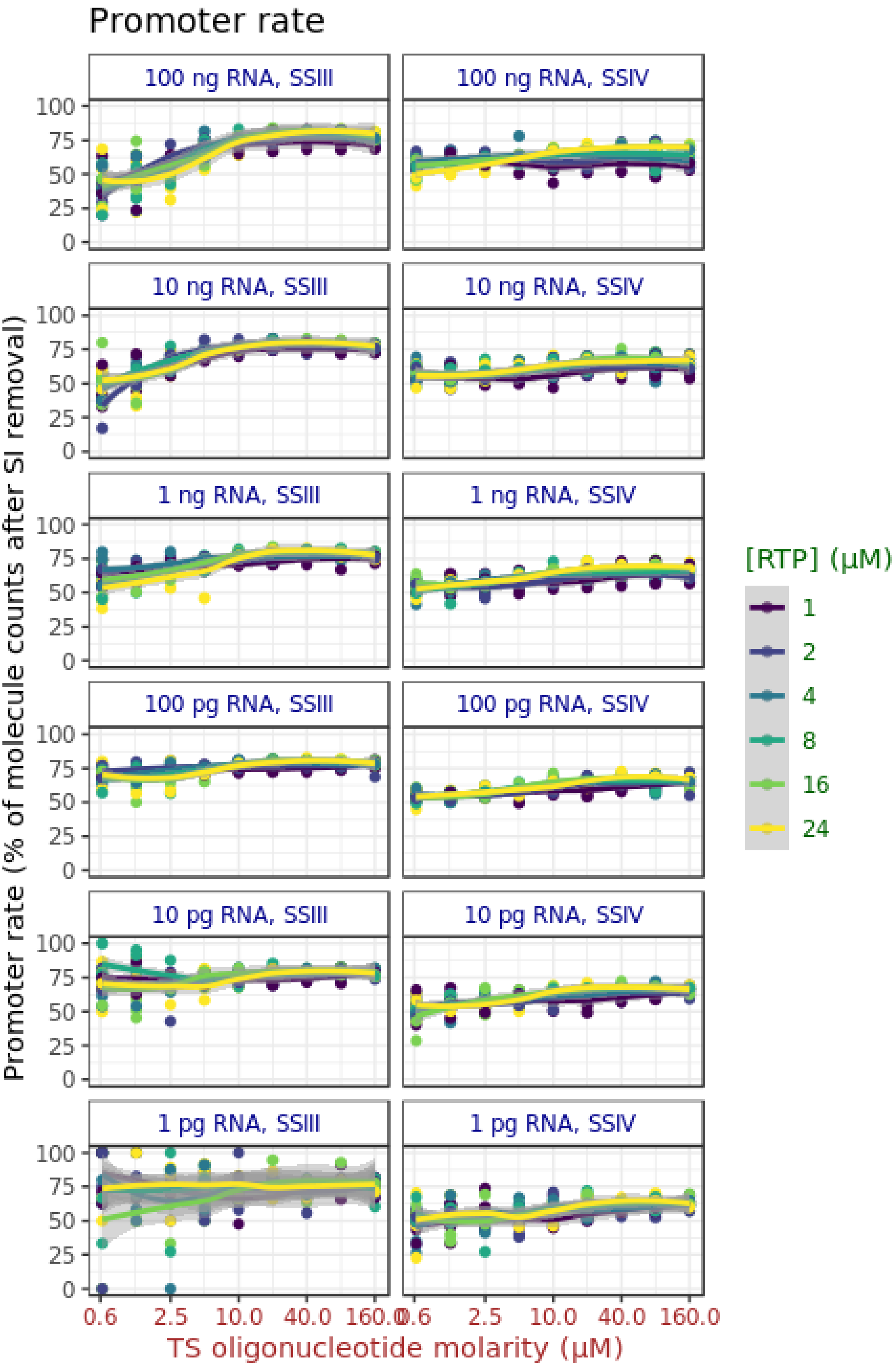

Promoter rates increases with TSO and RT primer molarity. It is higher with SuperScript III than with SSIV.

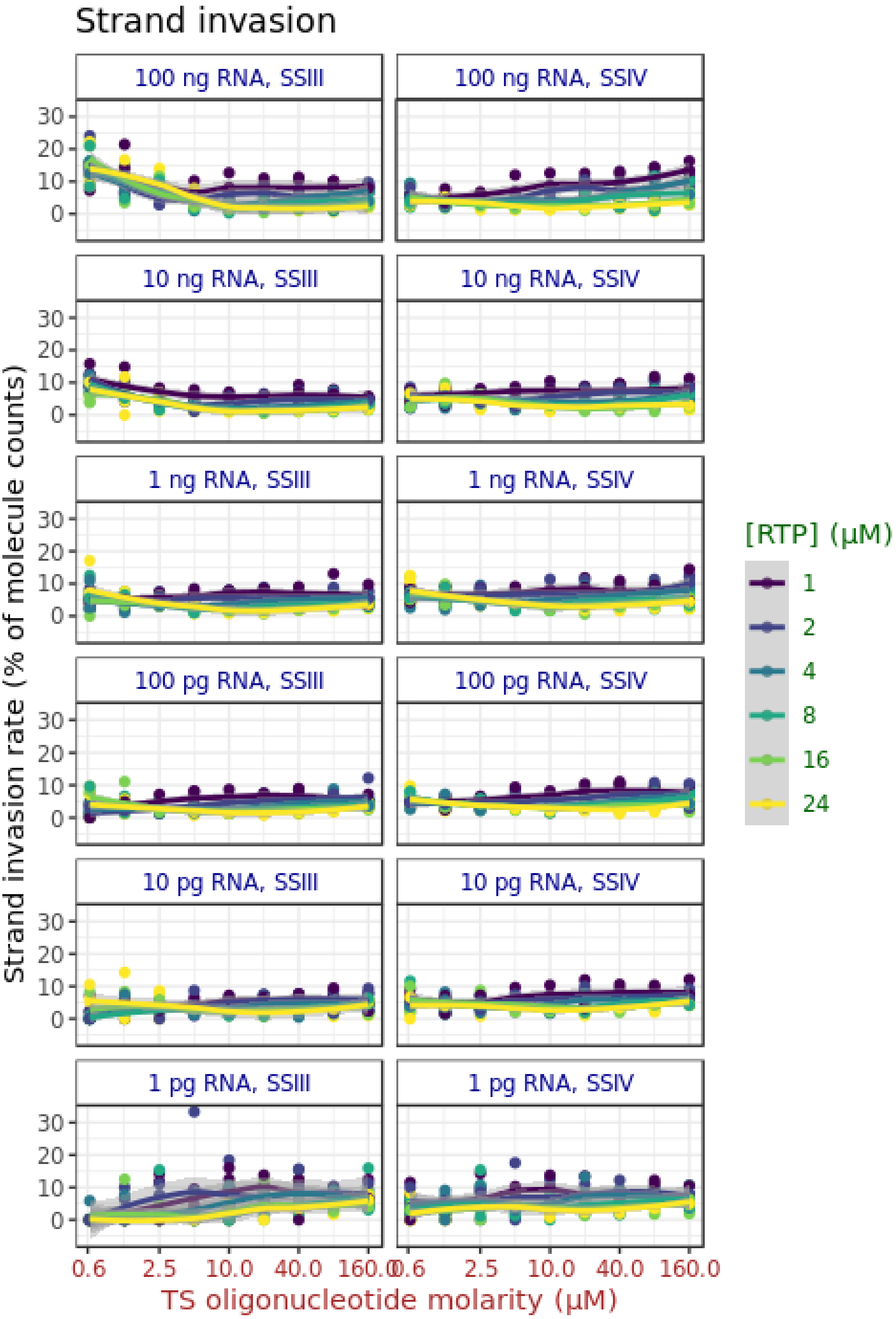

Strand invasion artefacts are reduced by adding more RT primers. With SuperScript III, the molarity of TSOs has to be increased at high RNA concentrations. This does not seem to be teh case with SuperScript IV.

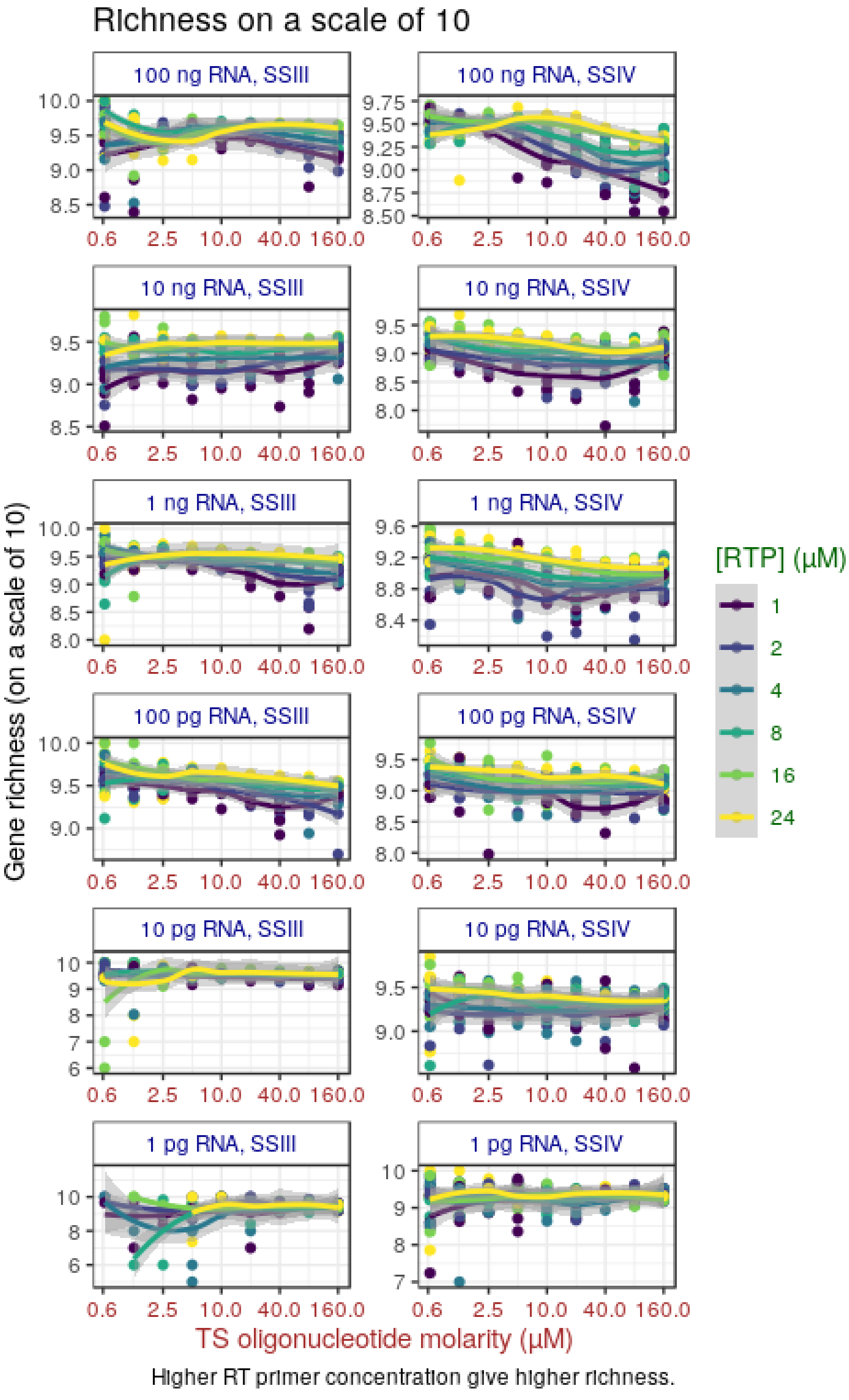

Richness on a scale of 10
100 ng RNA, SSIII
100 ng RNA, SSIV
10 ng RNA, SSIII
10 ng RNA, SSIV
1 ng RNA, SSIII
1 ng RNA, SSIV
100 pg RNA, SSIII
100 pg RNA, SSIV
10 pg RNA, SSIII
10 pg RNA, SSIV
1 pg RNA, SSIII
1 pg RNA, SSIV
Gene richness (on a scale of 10)
TS oligonucleotide molarity (µM)
0.6 2.5 10.0 40.0 160.0
[RTP] (µM)
1
2
4
8
16
24
Higher RT primer concentration give higher richness.

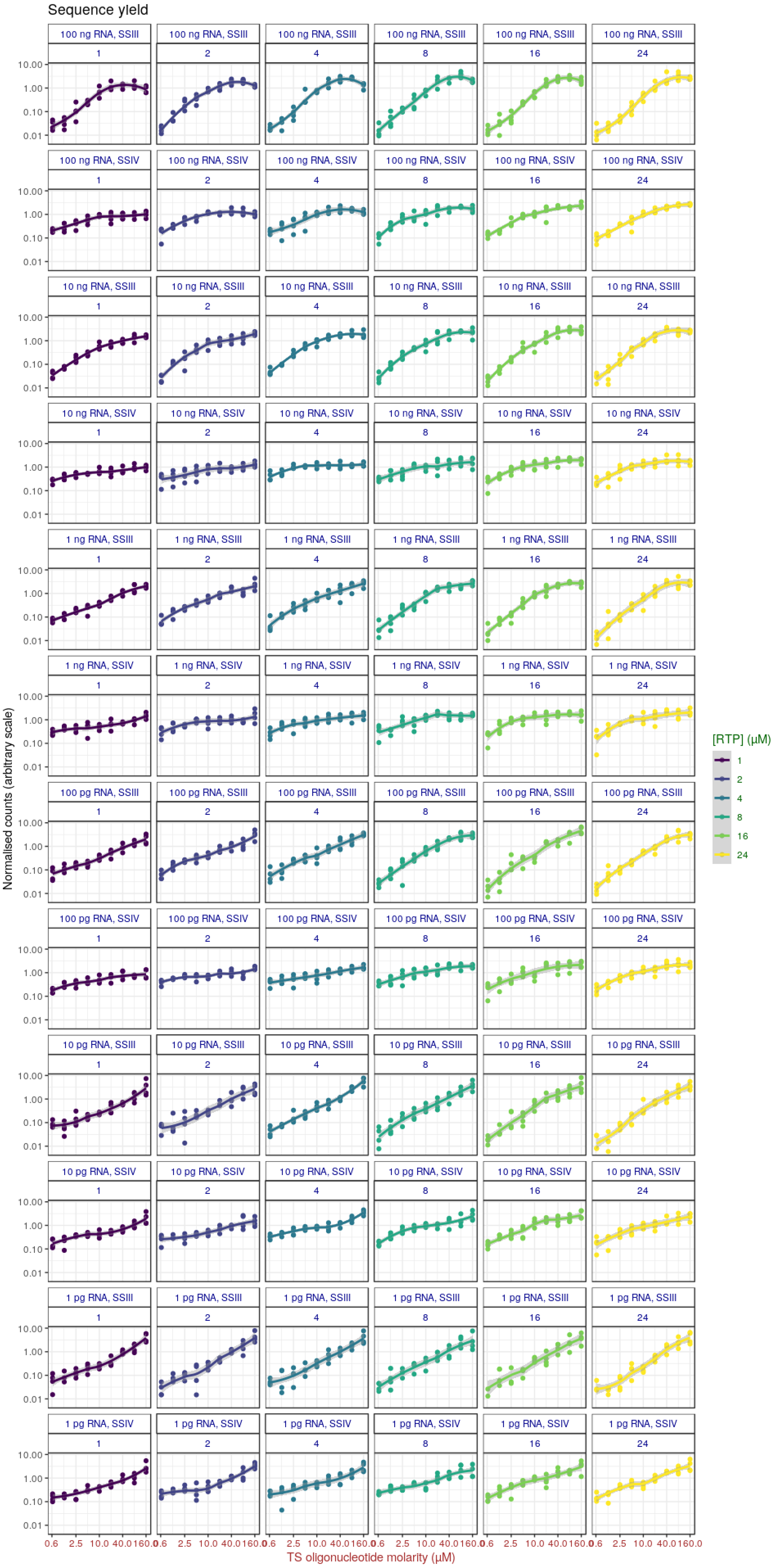
Sequence yield
100 ng RNA, SSIII
100 ng RNA, SSIV
10 ng RNA, SSIII
10 ng RNA, SSIV
1 ng RNA, SSIII
1 ng RNA, SSIV
100 pg RNA, SSIII
100 pg RNA, SSIV
10 pg RNA, SSIII
10 pg RNA, SSIV
1 pg RNA, SSIII
1 pg RNA, SSIV
1
2
4
8
16
24
10.00
1.00
0.10
0.01
0.6
2.5
10.0
40.0
160.0
Normalised counts (arbitrary scale)
TS oligonucleotide molarity (µM)
[RTP] (µM)
1
2
4
8
16
24

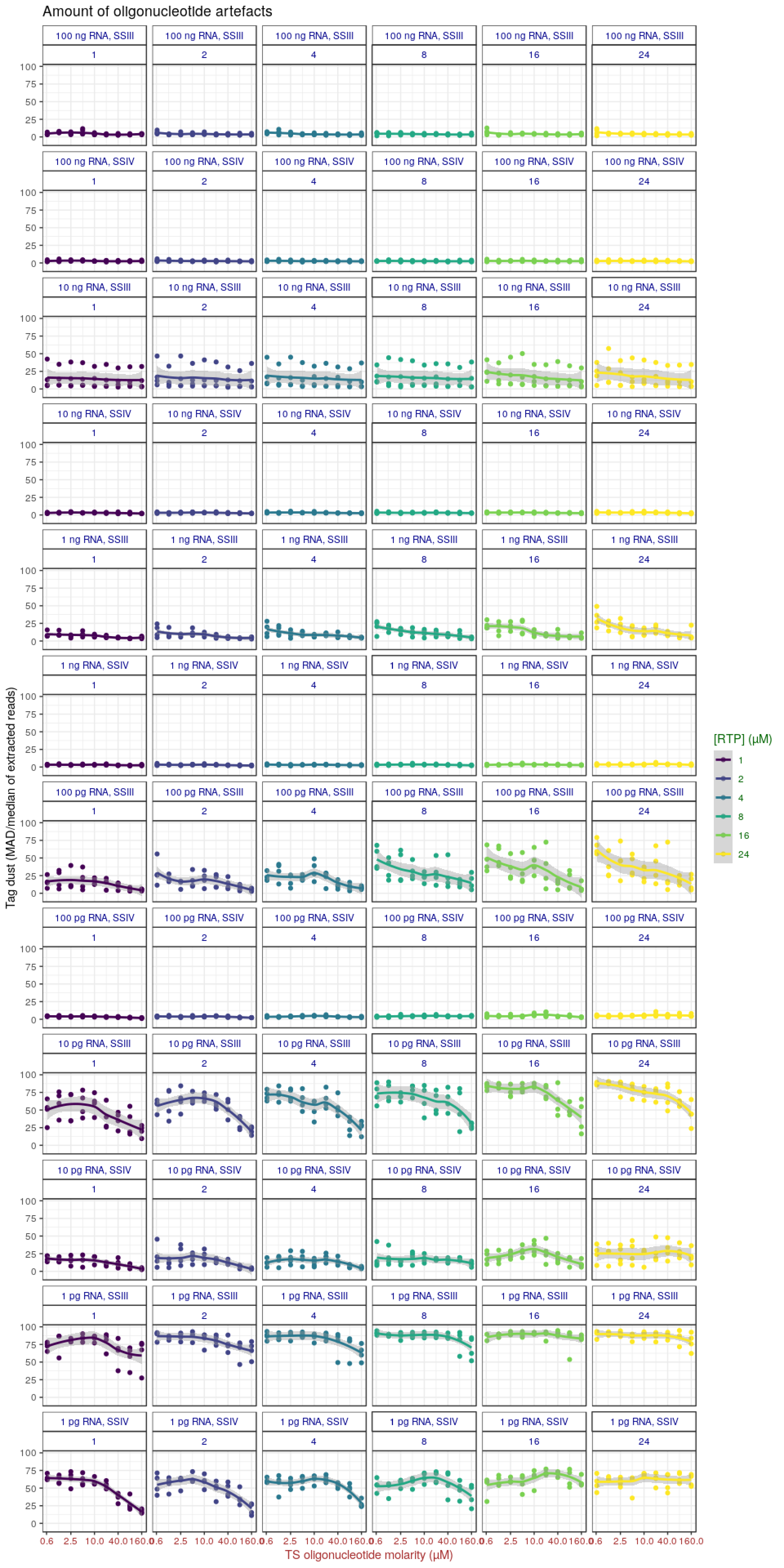

Amount of oligonucleotide artefacts
100 ng RNA, SSIII
100 ng RNA, SSIV
10 ng RNA, SSIII
10 ng RNA, SSIV
1 ng RNA, SSIII
1 ng RNA, SSIV
100 pg RNA, SSIII
100 pg RNA, SSIV
10 pg RNA, SSIII
10 pg RNA, SSIV
1 pg RNA, SSIII
1 pg RNA, SSIV
1
2
4
8
16
24
0
25
50
75
100
Tag dust (MAD/median of extracted reads)
0.6
2.5
10.0
40.0
160.0
TS oligonucleotide molarity (µM)
[RTP] (µM)
1
2
4
8
16
24

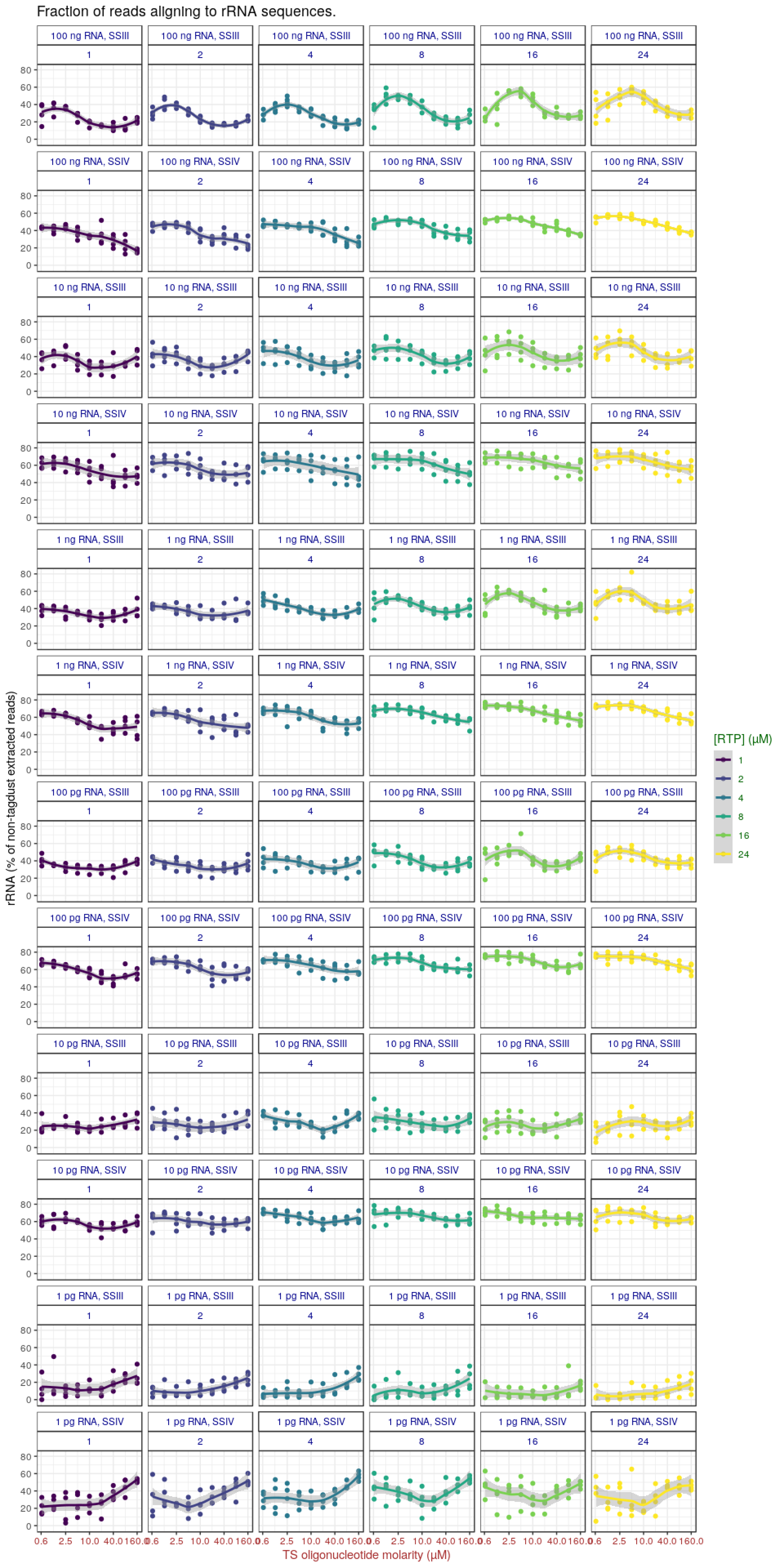
Fraction of reads aligning to rRNA sequences.
100 ng RNA, SSIII
100 ng RNA, SSIV
10 ng RNA, SSIII
10 ng RNA, SSIV
1 ng RNA, SSIII
1 ng RNA, SSIV
100 pg RNA, SSIII
100 pg RNA, SSIV
10 pg RNA, SSIII
10 pg RNA, SSIV
1 pg RNA, SSIII
1 pg RNA, SSIV
1
2
4
8
16
24
0
20
40
60
80
rRNA (% of non-tagdust extracted reads)
0.6
2.5
10.0
40.0
160.0
TS oligonucleotide molarity (µM)
[RTP] (µM)
1
2
4
8
16
24

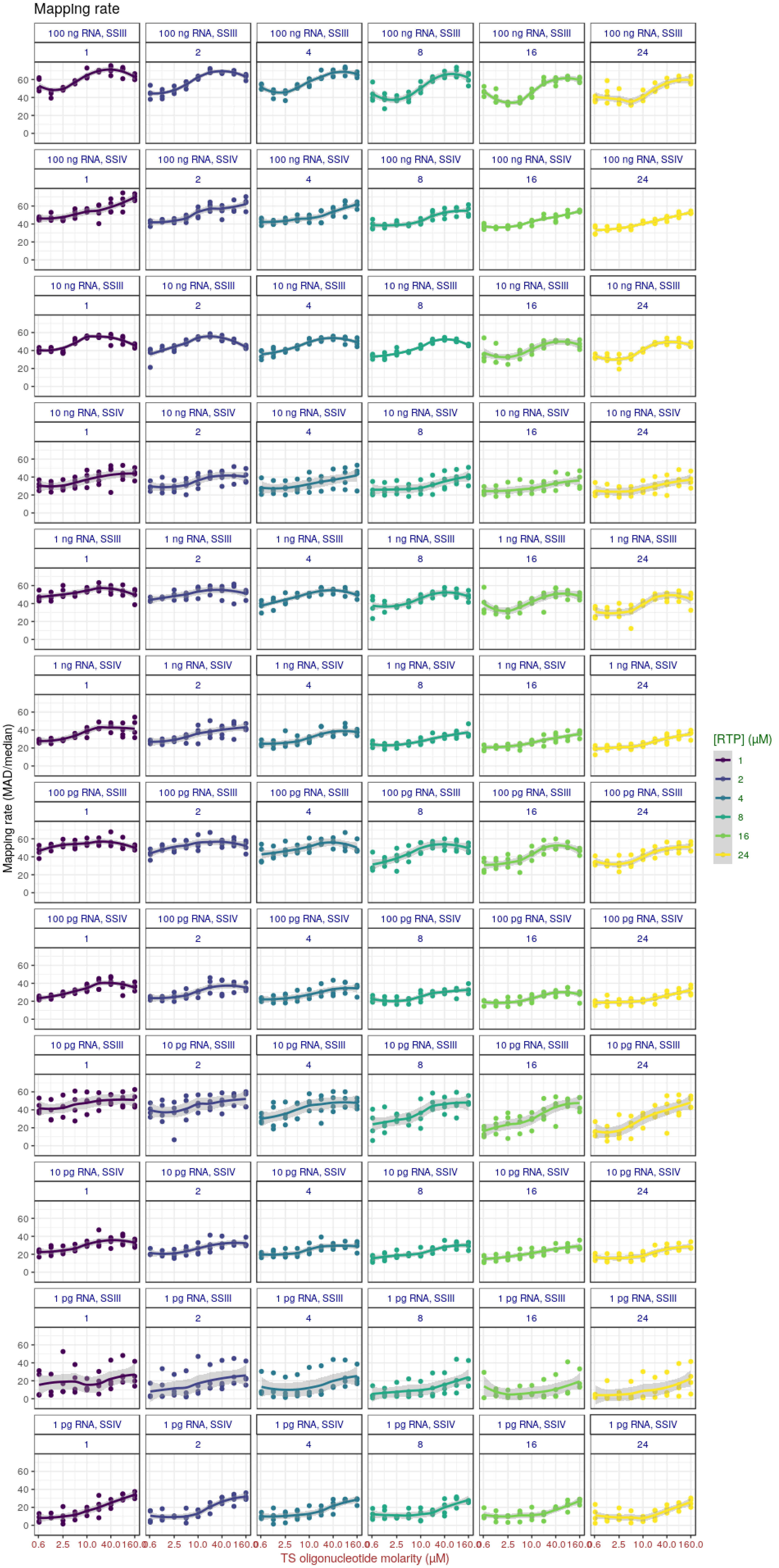
Mapping rate
100 ng RNA, SSIII
100 ng RNA, SSIV
10 ng RNA, SSIII
10 ng RNA, SSIV
1 ng RNA, SSIII
1 ng RNA, SSIV
100 pg RNA, SSIII
100 pg RNA, SSIV
10 pg RNA, SSIII
10 pg RNA, SSIV
1 pg RNA, SSIII
1 pg RNA, SSIV
1
2
4
8
16
24
Mapping rate (MAD/median)
0
20
40
60
0.6
2.5
10.0
40.0
160.0
TS oligonucleotide molarity (µM)
[RTP] (µM)
1
2
4
8
16
24

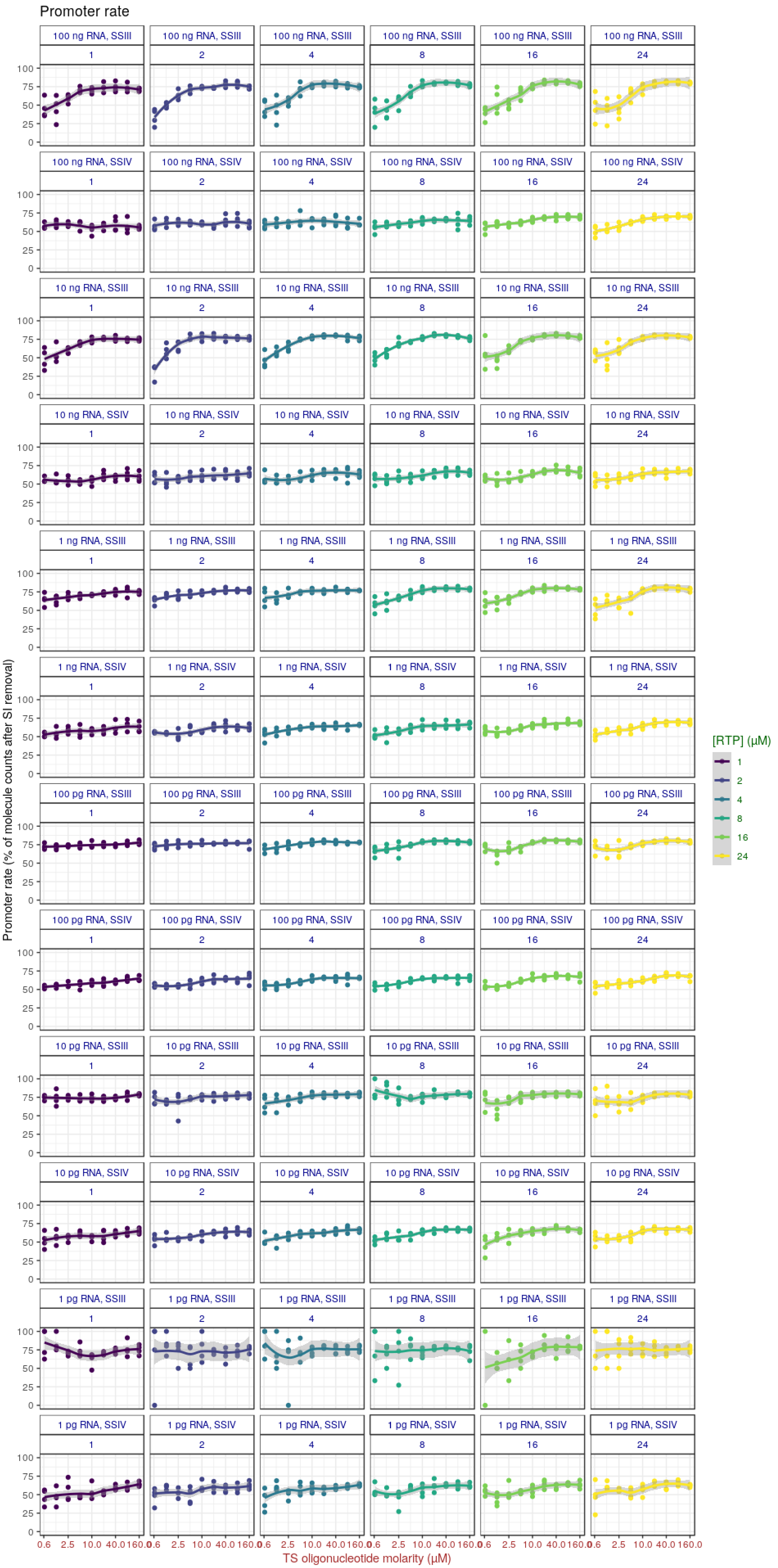
Promoter rate
Promoter rate (% of molecule counts after SI removal)
TS oligonucleotide molarity (µM)
[RTP] (µM)
1
2
4
8
16
24
100 ng RNA, SSIII
100 ng RNA, SSIV
10 ng RNA, SSIII
10 ng RNA, SSIV
1 ng RNA, SSIII
1 ng RNA, SSIV
100 pg RNA, SSIII
100 pg RNA, SSIV
10 pg RNA, SSIII
10 pg RNA, SSIV
1 pg RNA, SSIII
1 pg RNA, SSIV
0.6
2.5
10.0
40.0
160.0

Strand invasion

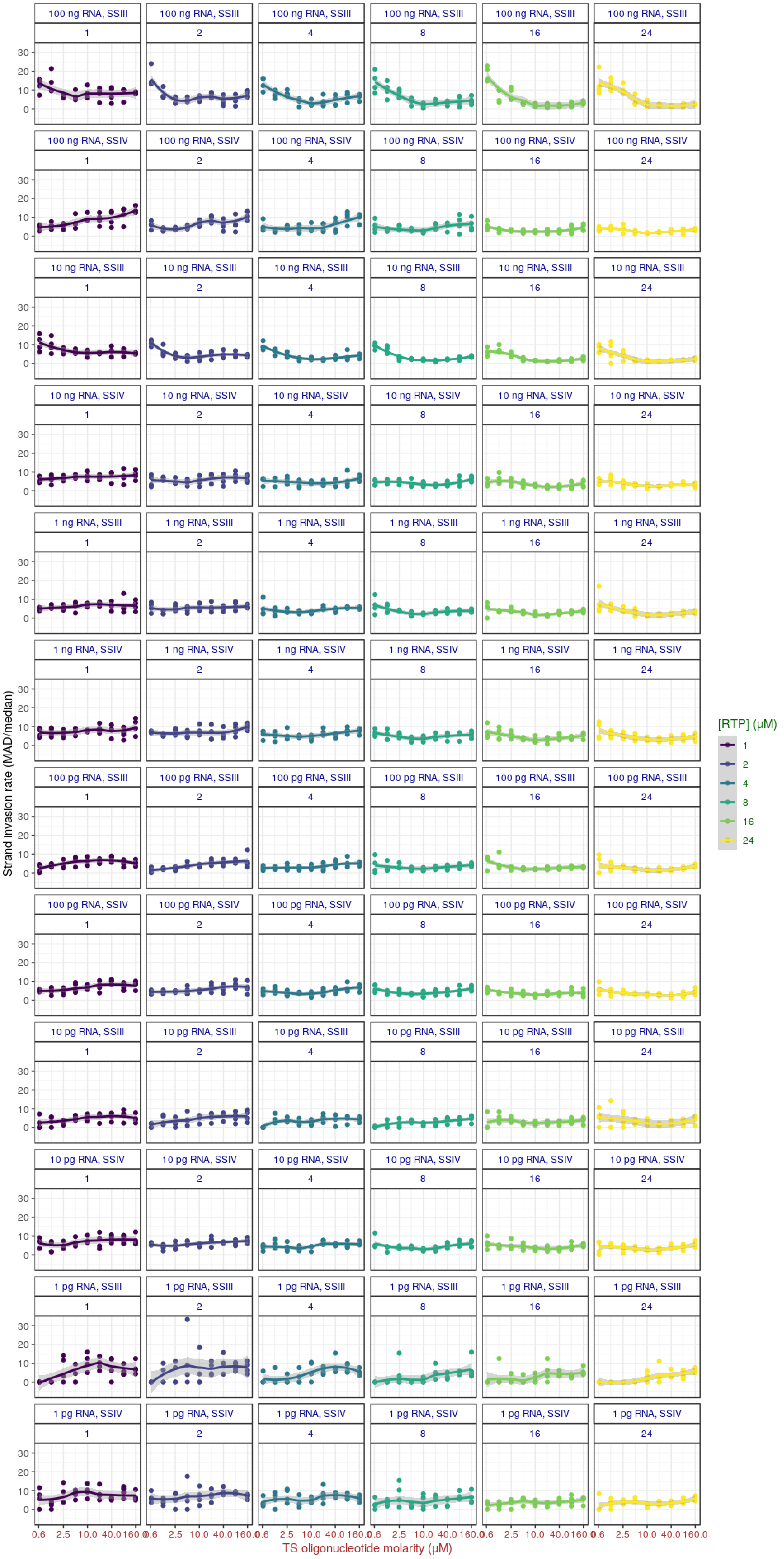

Richness on a scale of 10

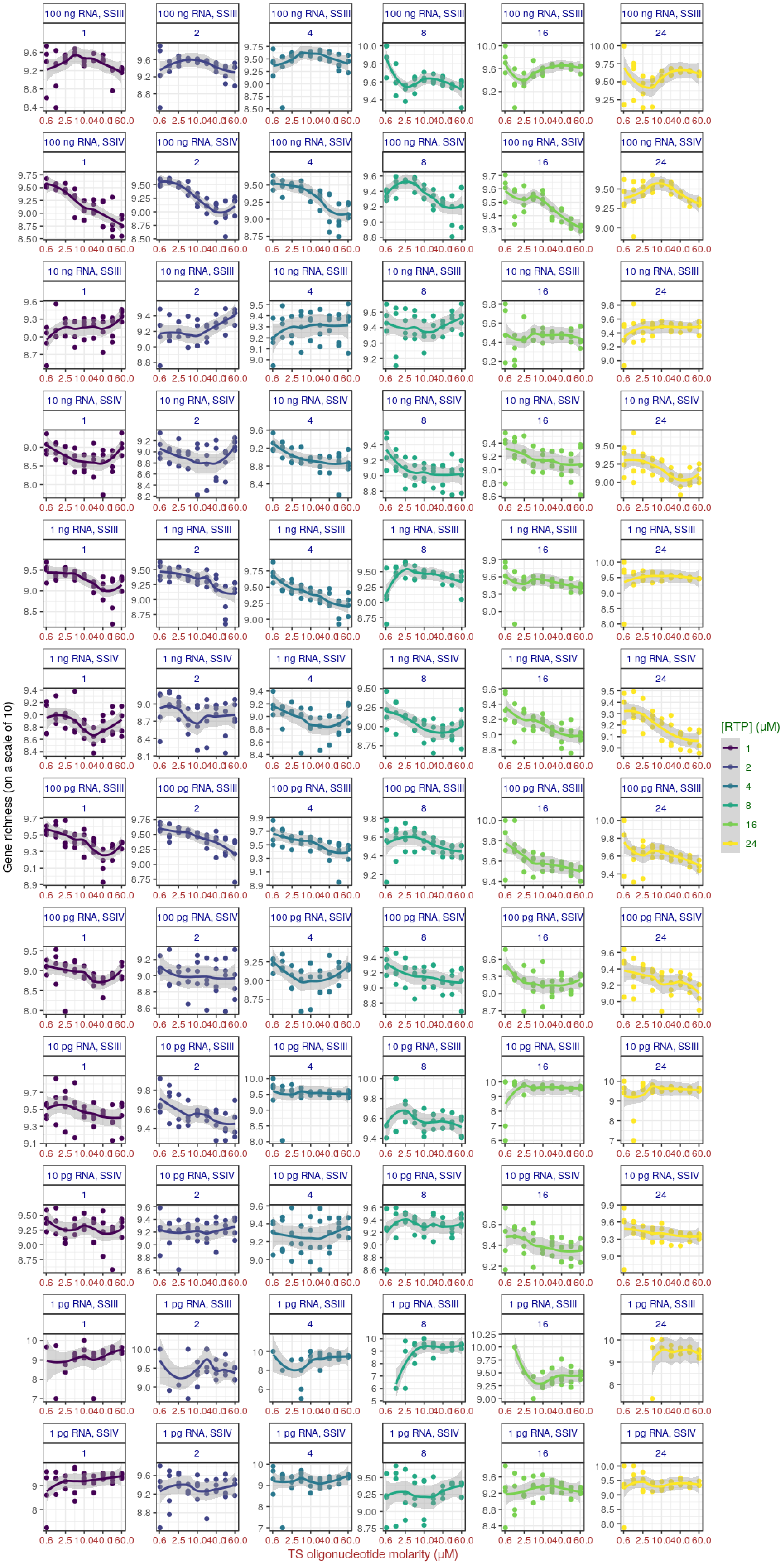

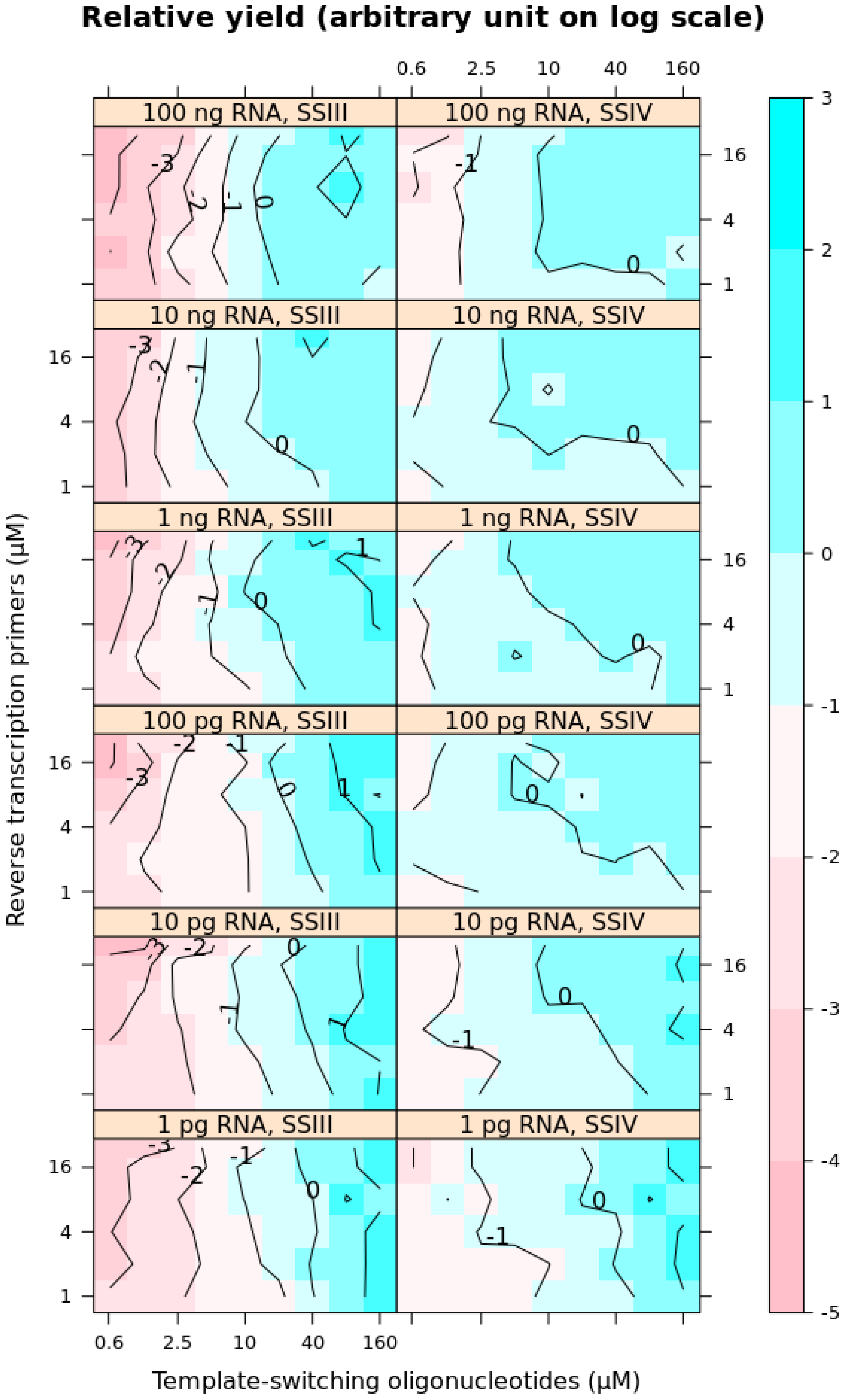

Relative yield (arbitrary unit on log scale)
100 ng RNA, SSIII
100 ng RNA, SSIV
10 ng RNA, SSIII
10 ng RNA, SSIV
1 ng RNA, SSIII
1 ng RNA, SSIV
100 pg RNA, SSIII
100 pg RNA, SSIV
10 pg RNA, SSIII
10 pg RNA, SSIV
1 pg RNA, SSIII
1 pg RNA, SSIV
0.6 2.5 10 40 160
1 4 16
3 2 1 0 -1 -2 -3 -4 -5
Reverse transcription primers (µM)
Template-switching oligonucleotides (µM)

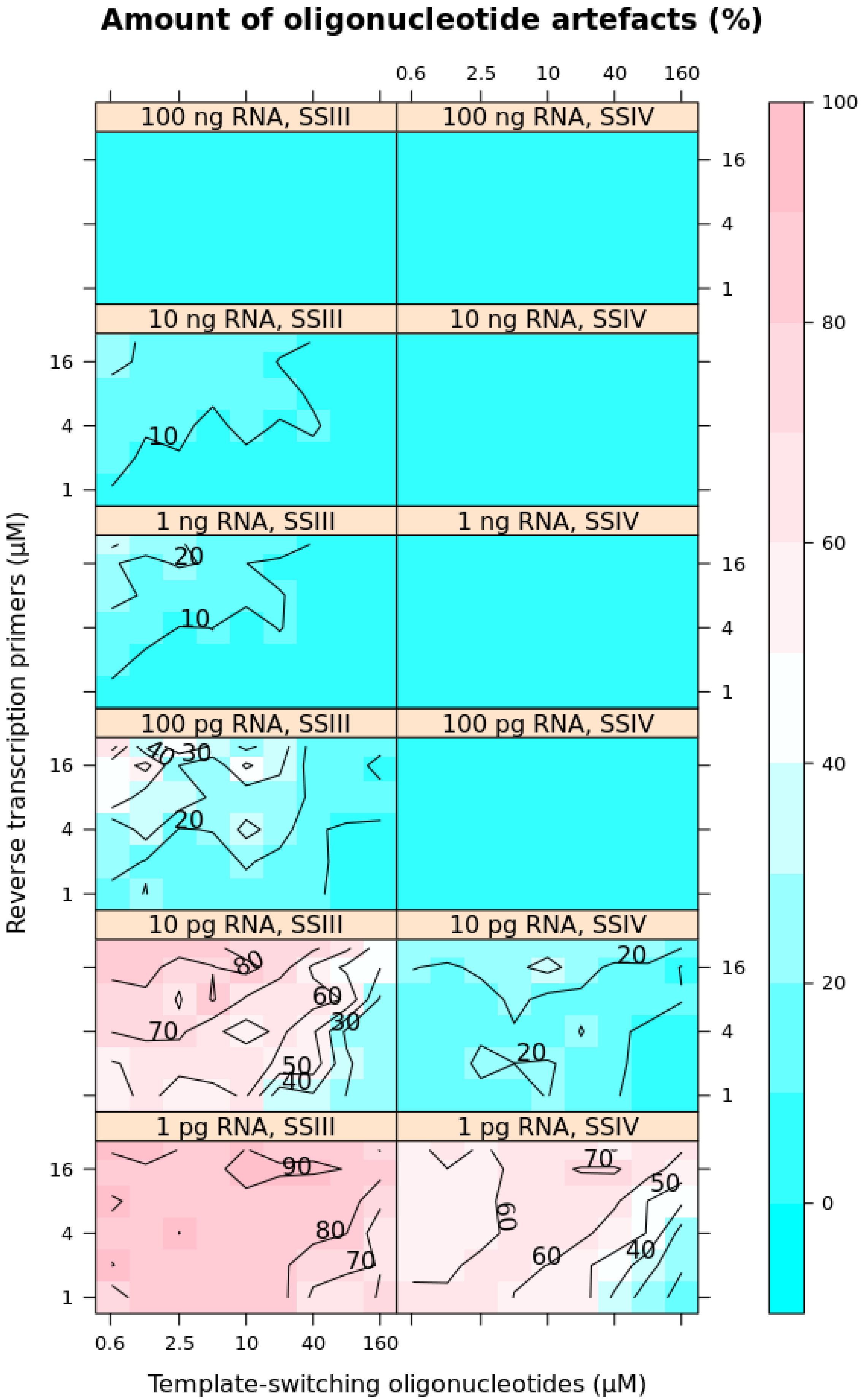

Amount of oligonucleotide artefacts (%)
100 ng RNA, SSIII
100 ng RNA, SSIV
10 ng RNA, SSIII
10 ng RNA, SSIV
1 ng RNA, SSIII
1 ng RNA, SSIV
100 pg RNA, SSIII
100 pg RNA, SSIV
10 pg RNA, SSIII
10 pg RNA, SSIV
1 pg RNA, SSIII
1 pg RNA, SSIV
Reverse transcription primers (µM)
Template-switching oligonucleotides (µM)
0.6
2.5
10
40
160
1
4
16
0
20
40
60
80
100

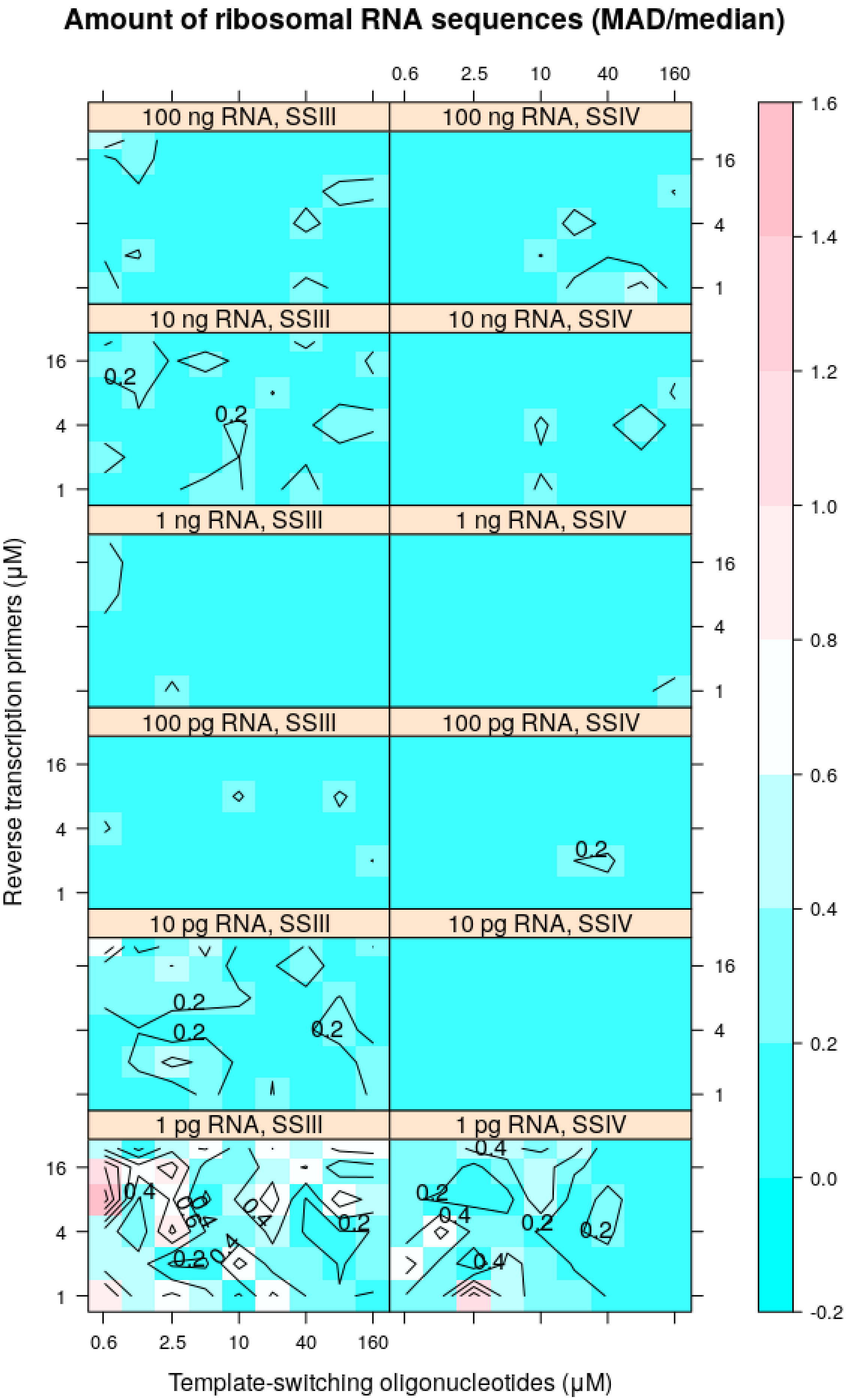
Amount of ribosomal RNA sequences (MAD/median)
100 ng RNA, SSIII
100 ng RNA, SSIV
10 ng RNA, SSIII
10 ng RNA, SSIV
1 ng RNA, SSIII
1 ng RNA, SSIV
100 pg RNA, SSIII
100 pg RNA, SSIV
10 pg RNA, SSIII
10 pg RNA, SSIV
1 pg RNA, SSIII
1 pg RNA, SSIV
Reverse transcription primers (μM)
Template-switching oligonucleotides (μM)
0.6
2.5
10
40
160
1
4
16
-0.2
0.0
0.2
0.4
0.6
0.8
1.0
1.2
1.4
1.6

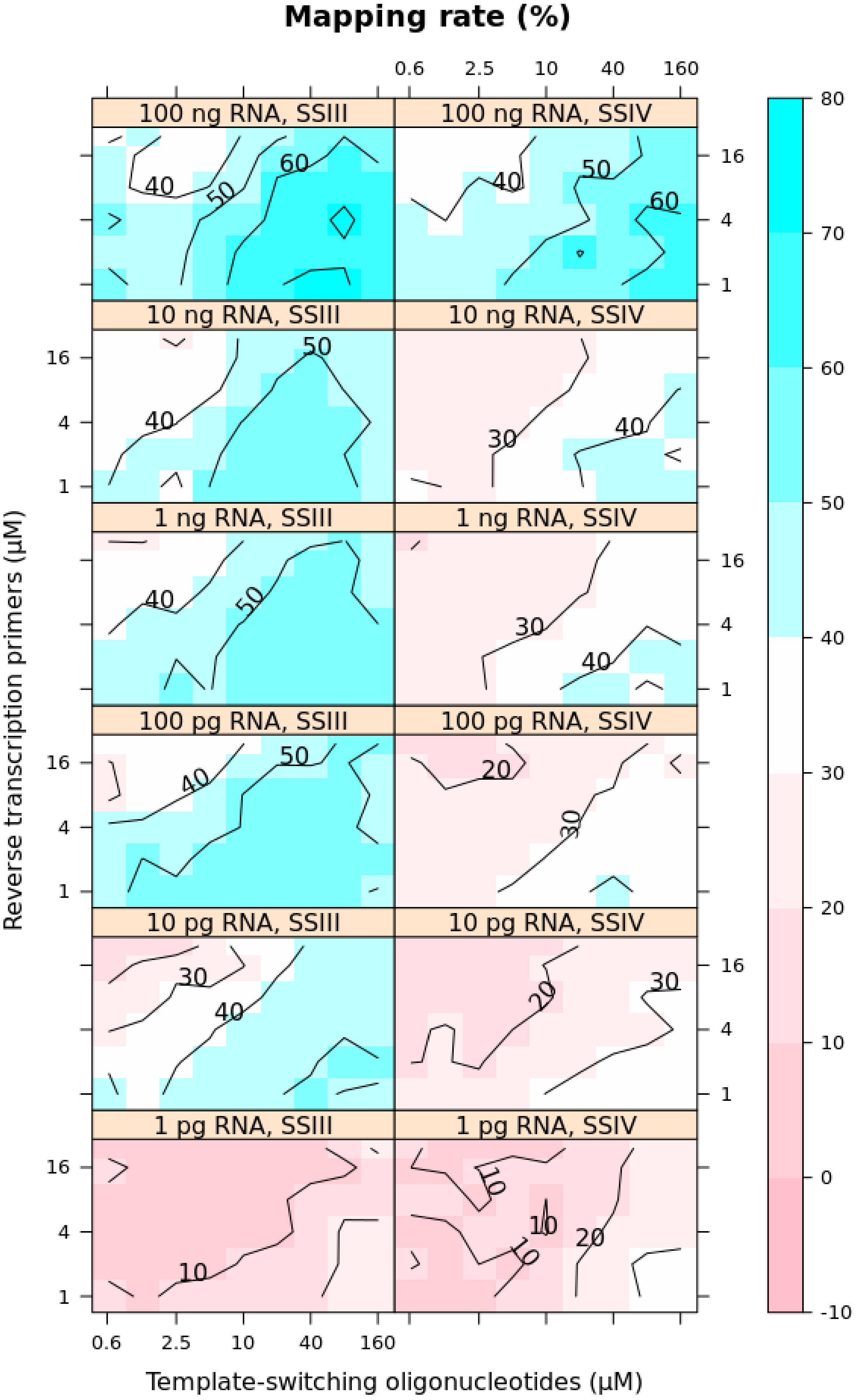

Mapping rate (%)
100 ng RNA, SSIII
100 ng RNA, SSIV
10 ng RNA, SSIII
10 ng RNA, SSIV
1 ng RNA, SSIII
1 ng RNA, SSIV
100 pg RNA, SSIII
100 pg RNA, SSIV
10 pg RNA, SSIII
10 pg RNA, SSIV
1 pg RNA, SSIII
1 pg RNA, SSIV
Reverse transcription primers (µM)
Template-switching oligonucleotides (µM)
0.6
2.5
10
40
160
1
4
16
-10
0
10
20
30
40
50
60
70
80

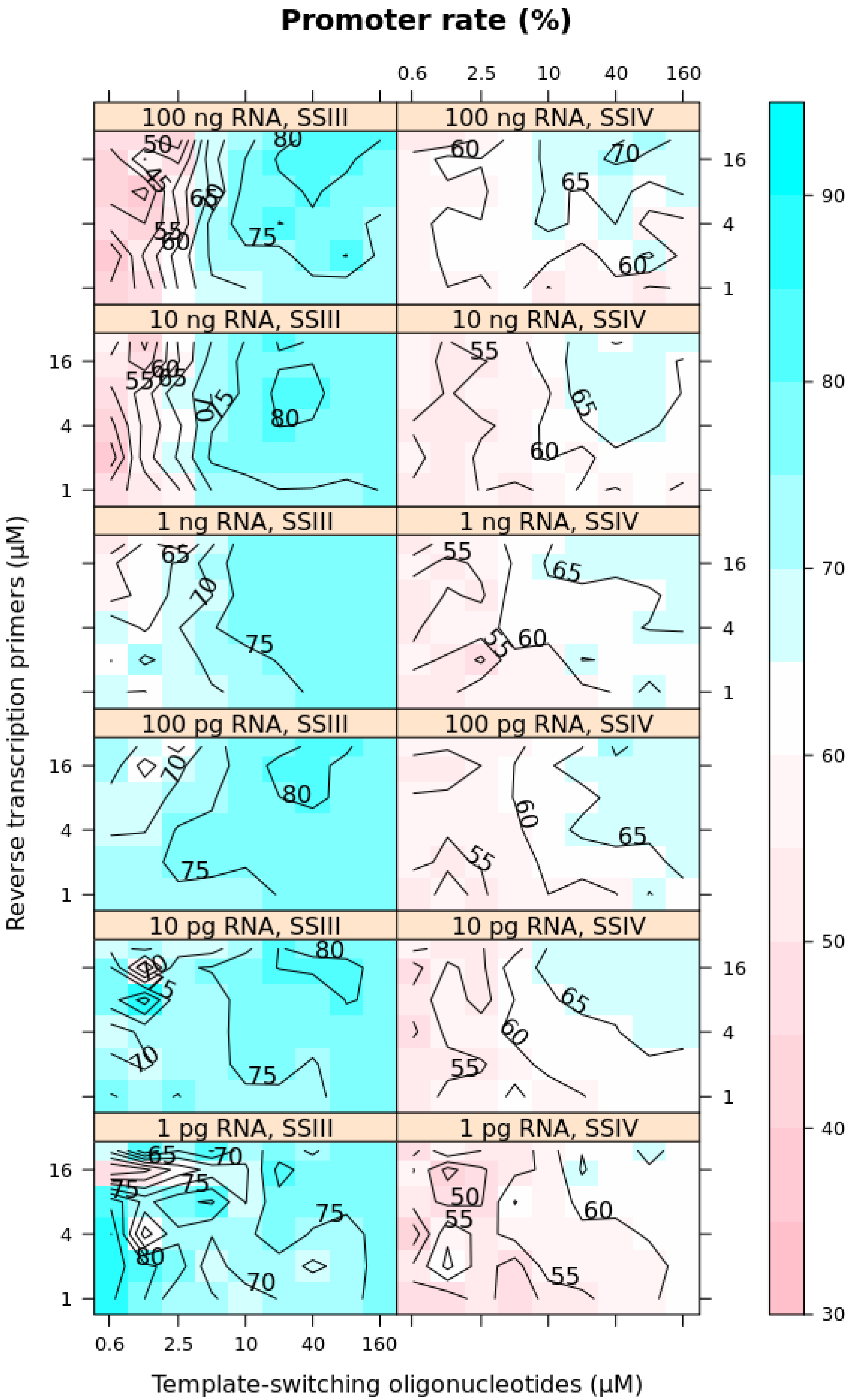

Promoter rate (%)
100 ng RNA, SSIII
100 ng RNA, SSIV
10 ng RNA, SSIII
10 ng RNA, SSIV
1 ng RNA, SSIII
1 ng RNA, SSIV
100 pg RNA, SSIII
100 pg RNA, SSIV
10 pg RNA, SSIII
10 pg RNA, SSIV
1 pg RNA, SSIII
1 pg RNA, SSIV
Reverse transcription primers (µM)
Template-switching oligonucleotides (µM)
0.6
2.5
10
40
160
1
4
16
30
40
50
60
70
80
90

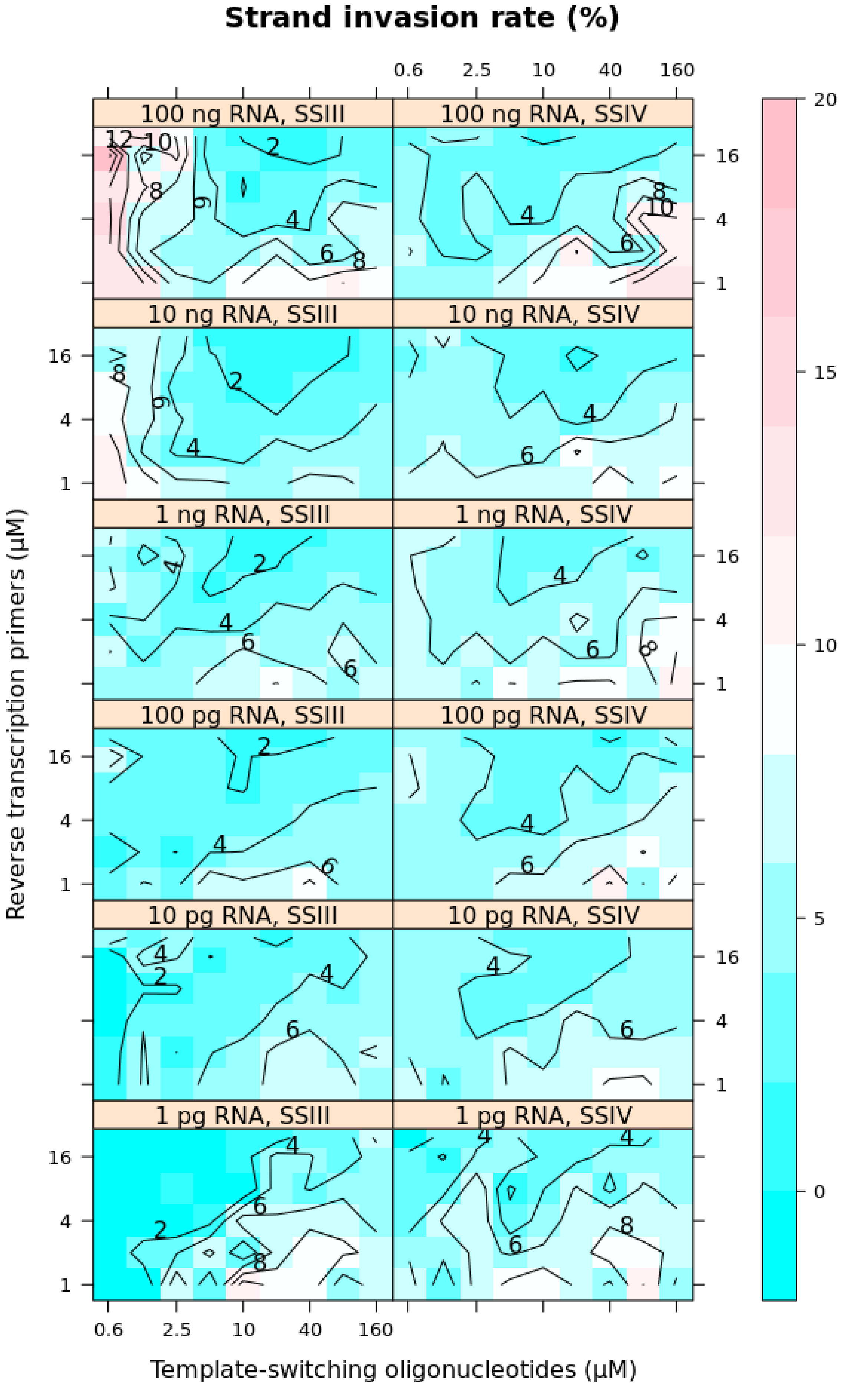

Strand invasion rate (%)
100 ng RNA, SSIII
100 ng RNA, SSIV
10 ng RNA, SSIII
10 ng RNA, SSIV
1 ng RNA, SSIII
1 ng RNA, SSIV
100 pg RNA, SSIII
100 pg RNA, SSIV
10 pg RNA, SSIII
10 pg RNA, SSIV
1 pg RNA, SSIII
1 pg RNA, SSIV
Reverse transcription primers (µM)
Template-switching oligonucleotides (µM)
0.6
2.5
10
40
160
1
4
16
0
5
10
15
20

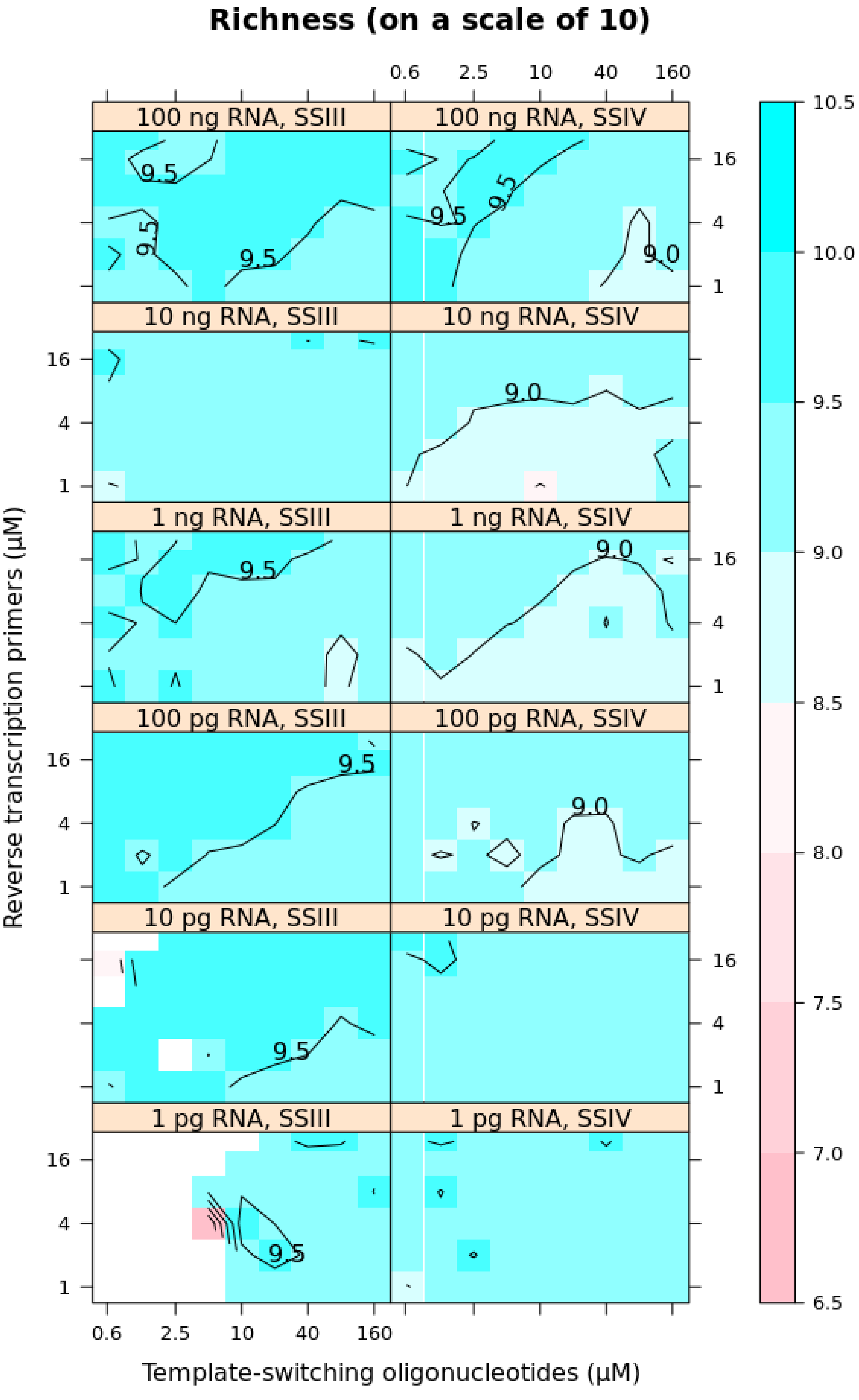
Richness (on a scale of 10)
100 ng RNA, SSIII
100 ng RNA, SSIV
10 ng RNA, SSIII
10 ng RNA, SSIV
1 ng RNA, SSIII
1 ng RNA, SSIV
100 pg RNA, SSIII
100 pg RNA, SSIV
10 pg RNA, SSIII
10 pg RNA, SSIV
1 pg RNA, SSIII
1 pg RNA, SSIV
Reverse transcription primers (µM)
Template-switching oligonucleotides (µM)
0.6
2.5
10
40
160
1
4
16
6.5
7.0
7.5
8.0
8.5
9.0
9.5
10.0
10.5

Relative yield (MAD/median)

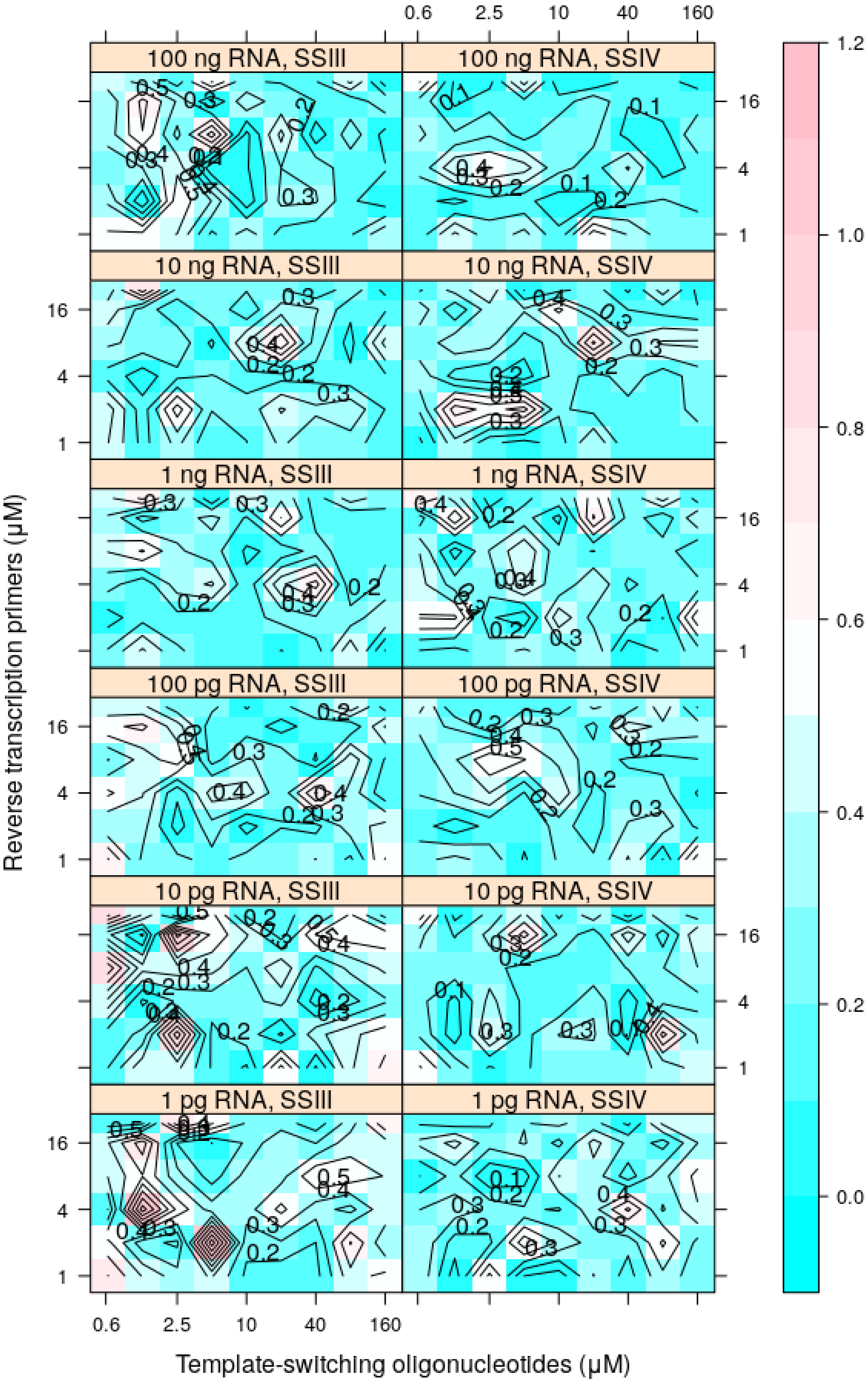

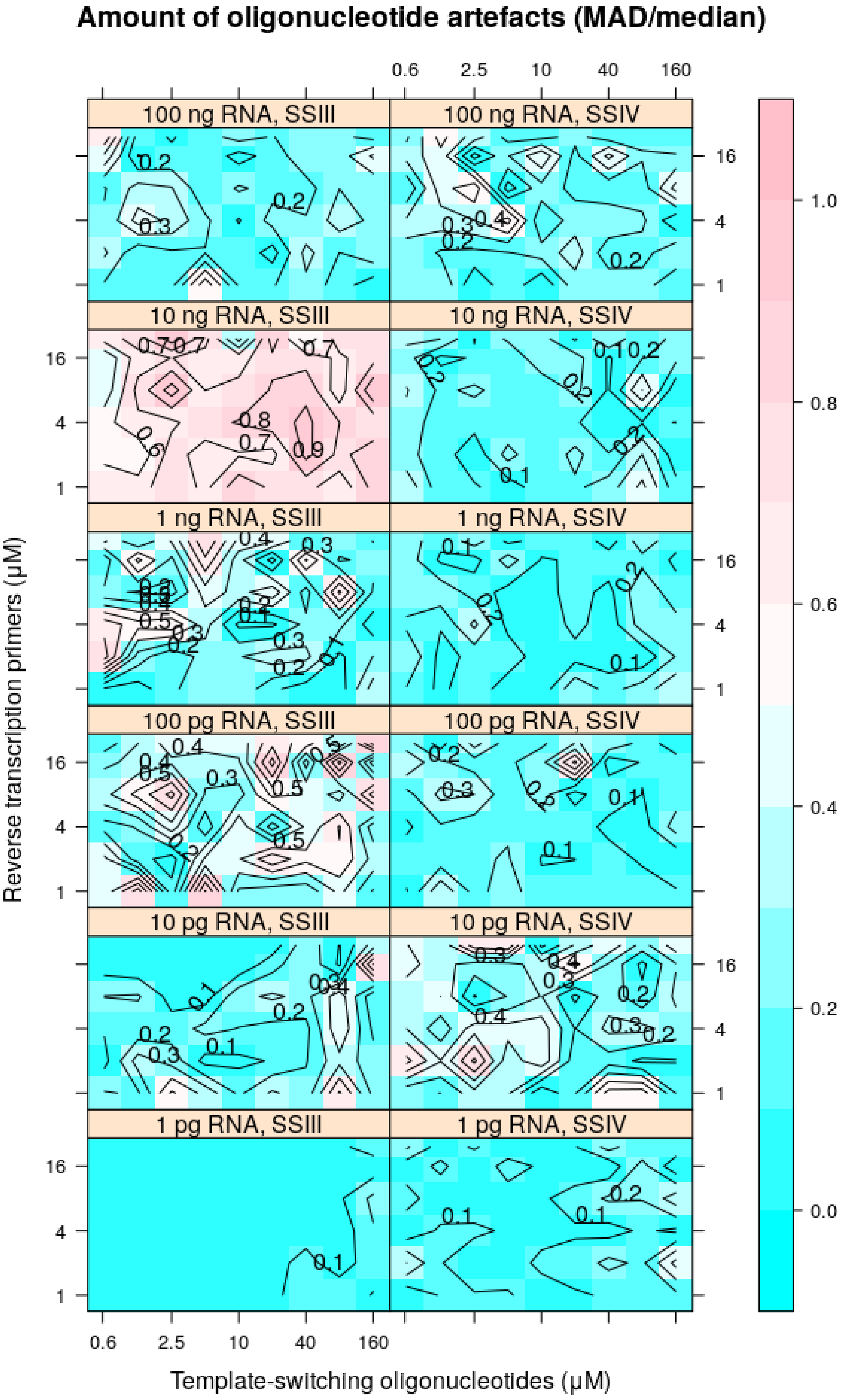
Amount of oligonucleotide artefacts (MAD/median)
100 ng RNA, SSIII
100 ng RNA, SSIV
10 ng RNA, SSIII
10 ng RNA, SSIV
1 ng RNA, SSIII
1 ng RNA, SSIV
100 pg RNA, SSIII
100 pg RNA, SSIV
10 pg RNA, SSIII
10 pg RNA, SSIV
1 pg RNA, SSIII
1 pg RNA, SSIV
Reverse transcription primers (µM)
Template-switching oligonucleotides (µM)
0.6 2.5 10 40 160
1 4 16
0.0 0.2 0.4 0.6 0.8 1.0

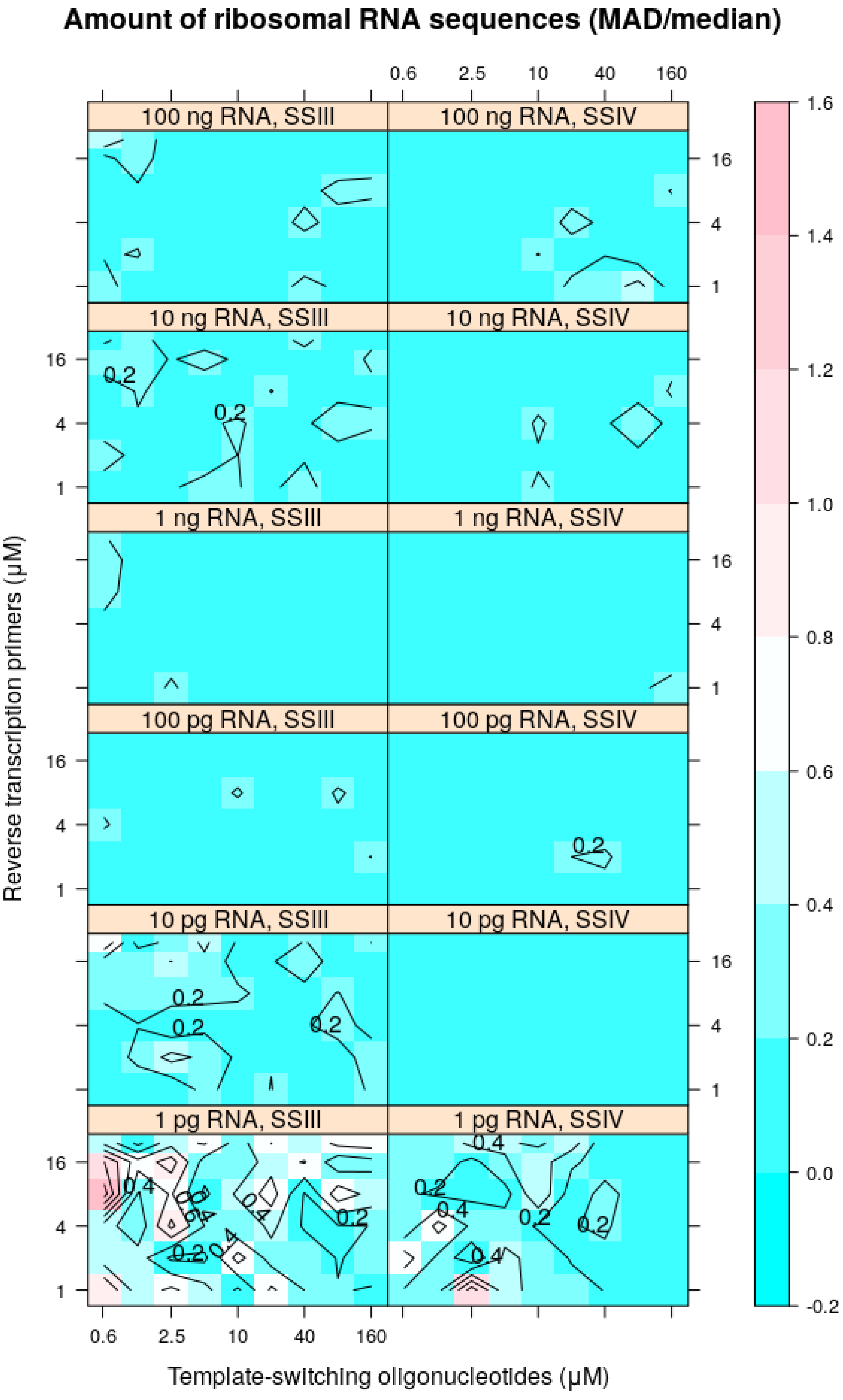

Amount of ribosomal RNA sequences (MAD/median)
100 ng RNA, SSIII
100 ng RNA, SSIV
10 ng RNA, SSIII
10 ng RNA, SSIV
1 ng RNA, SSIII
1 ng RNA, SSIV
100 pg RNA, SSIII
100 pg RNA, SSIV
10 pg RNA, SSIII
10 pg RNA, SSIV
1 pg RNA, SSIII
1 pg RNA, SSIV
Reverse transcription primers (μM)
Template-switching oligonucleotides (μM)
0.6
2.5
10
40
160
1
4
16
0.2
0.4
-0.2
0.0
0.6
0.8
1.0
1.2
1.4
1.6

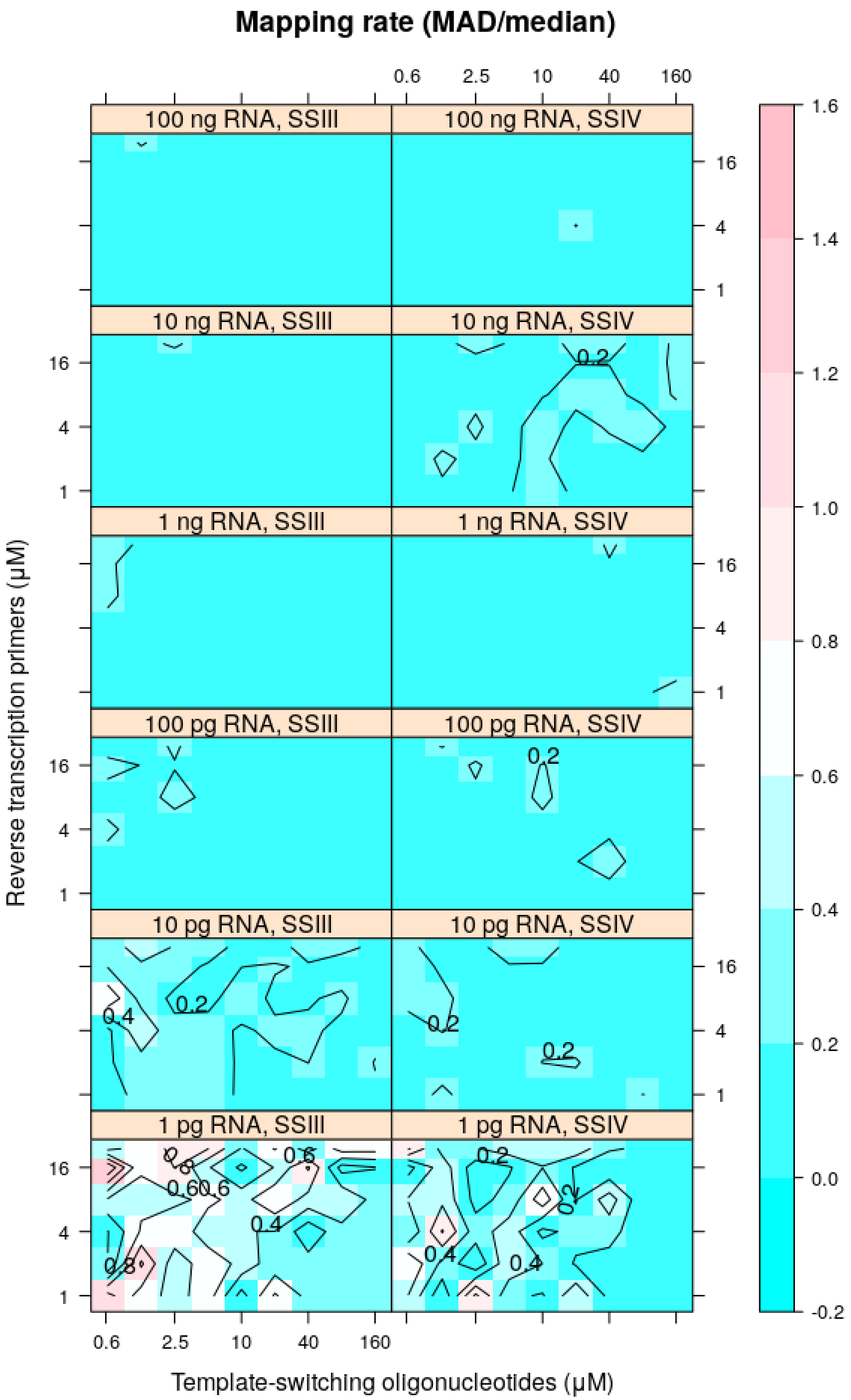
Mapping rate (MAD/median)
100 ng RNA, SSIII
100 ng RNA, SSIV
10 ng RNA, SSIII
10 ng RNA, SSIV
1 ng RNA, SSIII
1 ng RNA, SSIV
100 pg RNA, SSIII
100 pg RNA, SSIV
10 pg RNA, SSIII
10 pg RNA, SSIV
1 pg RNA, SSIII
1 pg RNA, SSIV
Reverse transcription primers (µM)
Template-switching oligonucleotides (µM)
0.6
2.5
10
40
160
1
4
16
-0.2
0.0
0.2
0.4
0.6
0.8
1.0
1.2
1.4
1.6

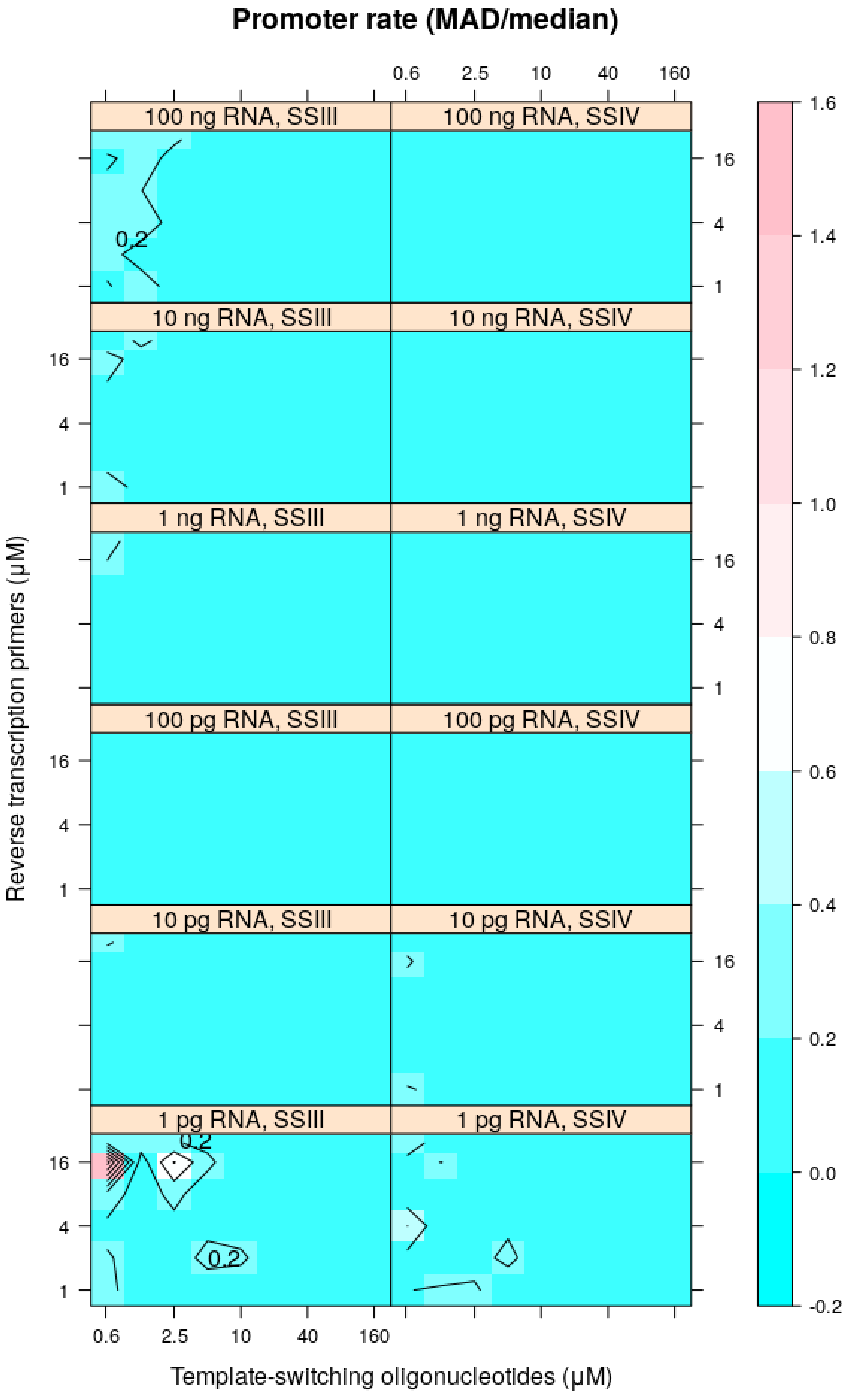
Promoter rate (MAD/median)
100 ng RNA, SSIII
100 ng RNA, SSIV
10 ng RNA, SSIII
10 ng RNA, SSIV
1 ng RNA, SSIII
1 ng RNA, SSIV
100 pg RNA, SSIII
100 pg RNA, SSIV
10 pg RNA, SSIII
10 pg RNA, SSIV
1 pg RNA, SSIII
1 pg RNA, SSIV
0.2
0.6
2.5
10
40
160
16
4
1
1.6
1.4
1.2
1.0
0.8
0.6
0.4
0.2
0.0
-0.2
Reverse transcription primers (µM)
Template-switching oligonucleotides (µM)

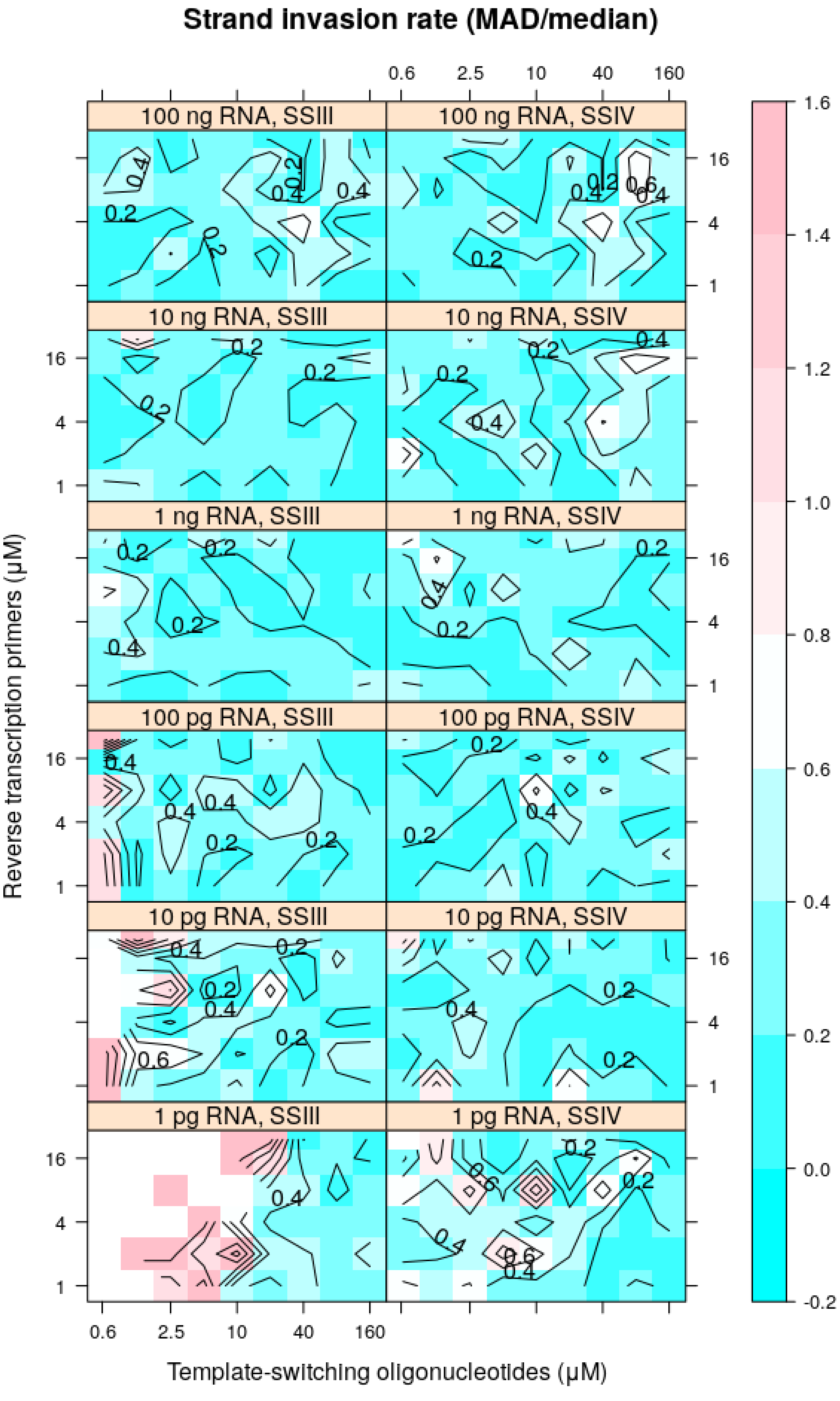

Strand invasion rate (MAD/median)
100 ng RNA, SSIII
100 ng RNA, SSIV
10 ng RNA, SSIII
10 ng RNA, SSIV
1 ng RNA, SSIII
1 ng RNA, SSIV
100 pg RNA, SSIII
100 pg RNA, SSIV
10 pg RNA, SSIII
10 pg RNA, SSIV
1 pg RNA, SSIII
1 pg RNA, SSIV
Reverse transcription primers (µM)
Template-switching oligonucleotides (µM)
0.6
2.5
10
40
160
1
4
16
-0.2
0.0
0.2
0.4
0.6
0.8
1.0
1.2
1.4
1.6

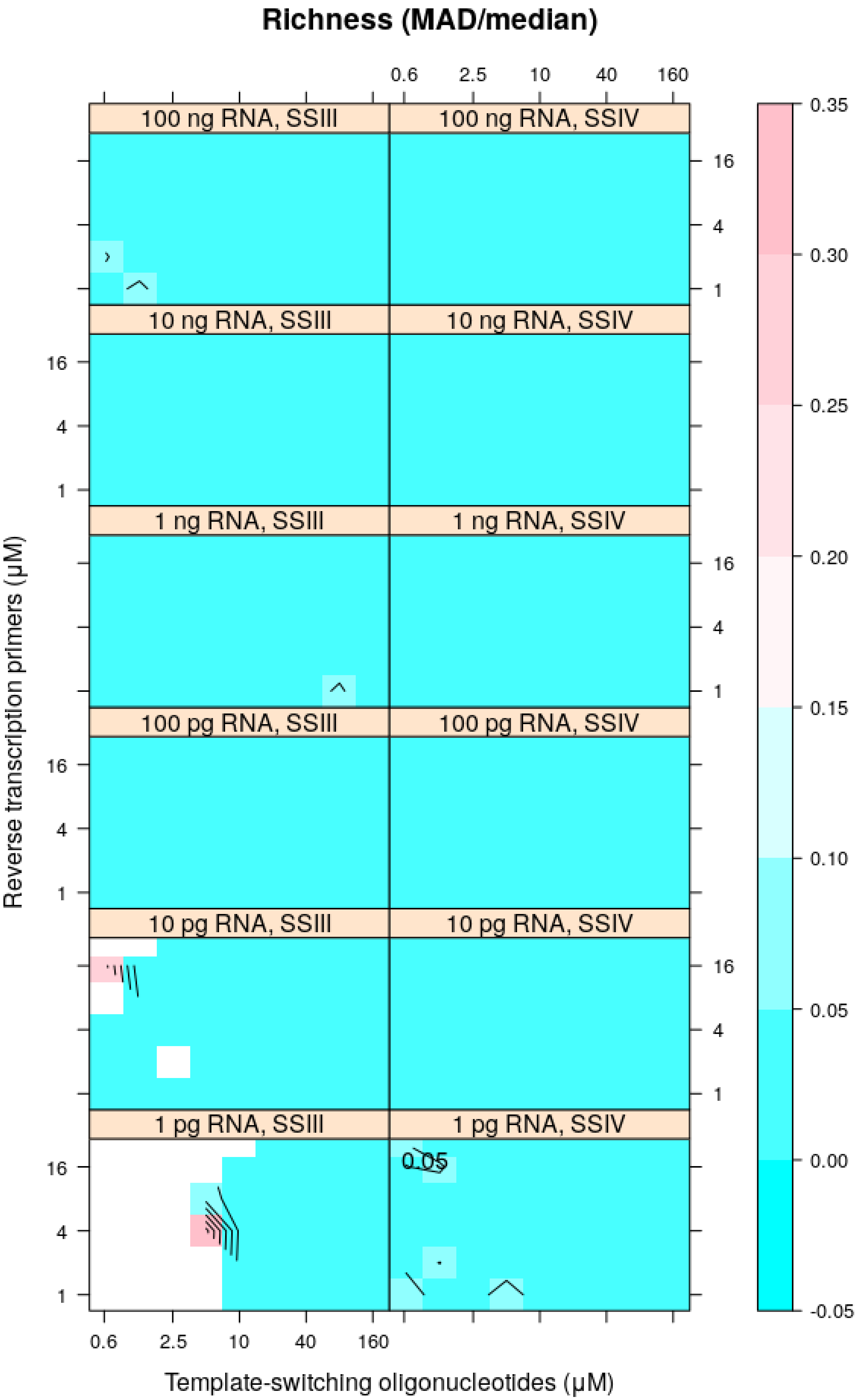

Richness (MAD/median)
100 ng RNA, SSIII
100 ng RNA, SSIV
10 ng RNA, SSIII
10 ng RNA, SSIV
1 ng RNA, SSIII
1 ng RNA, SSIV
100 pg RNA, SSIII
100 pg RNA, SSIV
10 pg RNA, SSIII
10 pg RNA, SSIV
1 pg RNA, SSIII
1 pg RNA, SSIV
0.05
0.6
2.5
10
40
160
16
4
1
0.35
0.30
0.25
0.20
0.15
0.10
0.05
0.00
-0.05
Reverse transcription primers (μM)
Template-switching oligonucleotides (μM)
